# Identification of 8-Hydroxyquinoline Derivatives That Decrease Cystathionine Beta Synthase (CBS) Activity

**DOI:** 10.3390/ijms23126769

**Published:** 2022-06-17

**Authors:** Pierre Conan, Alice Léon, Mathilde Gourdel, Claire Rollet, Loubna Chaïr, Noéline Caroff, Nelig Le Goux, Catherine Le Jossic-Corcos, Maha Sinane, Lucile Gentile, Louise Maillebouis, Nadège Loaëc, Jennifer Martin, Marie Vilaire, Laurent Corcos, Olivier Mignen, Mikael Croyal, Cécile Voisset, Frédéric Bihel, Gaëlle Friocourt

**Affiliations:** 1 Inserm, Université de Brest, EFS, UMR 1078, GGB, 29200 Brest, France; pierre.conan@univ-brest.fr (P.C.); alice.leon10@gmail.com (A.L.); claire.rollet@univ-brest.fr (C.R.); loubna.chair@univ-angers.fr (L.C.); noeline.caroff@univ-rennes.fr (N.C.); catherine.corcos@univ-brest.fr (C.L.J.-C.); maha.sinane@univ-brest.fr (M.S.); lucile.gentile@univ-brest.fr (L.G.); nadege.loaec@univ-brest.fr (N.L.); jennifer.martin@univ-brest.fr (J.M.); laurent.corcos@univ-brest.fr (L.C.); cecile.voisset@univ-brest.fr (C.V.); 2 Inserm, Université de Nantes, CHU Nantes, CNRS, L’Institut Du Thorax, 44000 Nantes, France; mathilde.gourdel@univ-nantes.fr (M.G.); mikael.croyal@univ-nantes.fr (M.C.); 3 CRNH-Ouest Mass Spectrometry Core Facility, 44000 Nantes, France; 4 Inserm U1227, Lymphocytes B, Autoimmunité et Immunothérapies, Université de Brest, 29200 Brest, France; nelig.legoux@univ-brest.fr (N.L.G.); olivier.mignen@univ-brest.fr (O.M.); 5 CRB-Biojel, Institut Jérôme Lejeune, 75015 Paris, France; louise.maillebouis@institutlejeune.org (L.M.); marie.vilaire@institutlejeune.org (M.V.); 6 Inserm, Université de Nantes, CHU Nantes, CNRS, SFR Santé, Inserm UMS 016, CNRS UMS 3556, 44000 Nantes, France; 7 Laboratoire d’Innovation Thérapeutique, LIT, UMR7200, IMS MEDALIS, Faculty of Pharmacy, CNRS, Université de Strasbourg, 67400 Illkirch, France; frederic.bihel@unistra.fr

**Keywords:** CBS, drug screening, Cys4, copper, zinc, cytosolic pH, Gex1/Gex2

## Abstract

CBS encodes a pyridoxal 5′-phosphate-dependent enzyme that catalyses the condensation of homocysteine and serine to form cystathionine. Due to its implication in some cancers and in the cognitive pathophysiology of Down syndrome, the identification of pharmacological inhibitors of this enzyme is urgently required. However, thus far, attempts to identify such molecules have only led to the identification of compounds with low potency and limited selectivity. We consequently developed an original, yeast-based screening method that identified three FDA-approved drugs of the 8-hydroxyquinoline family: clioquinol, chloroxine and nitroxoline. These molecules reduce CBS enzymatic activity in different cellular models, proving that the molecular mechanisms involved in yeast phenotypic rescue are conserved in mammalian cells. A combination of genetic and chemical biology approaches also revealed the importance of copper and zinc intracellular levels in the regulation of CBS enzymatic activity—copper promoting CBS activity and zinc inhibiting its activity. Taken together, these results indicate that our effective screening approach identified three new potent CBS inhibitors and provides new findings for the regulation of CBS activity, which is crucial to develop new therapies for CBS-related human disorders.

## 1. Introduction

**Figure 1:**
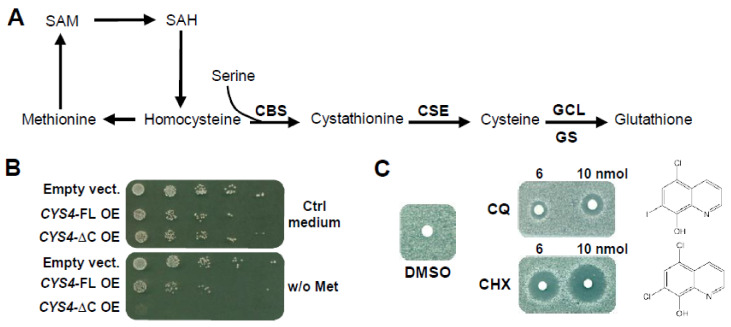

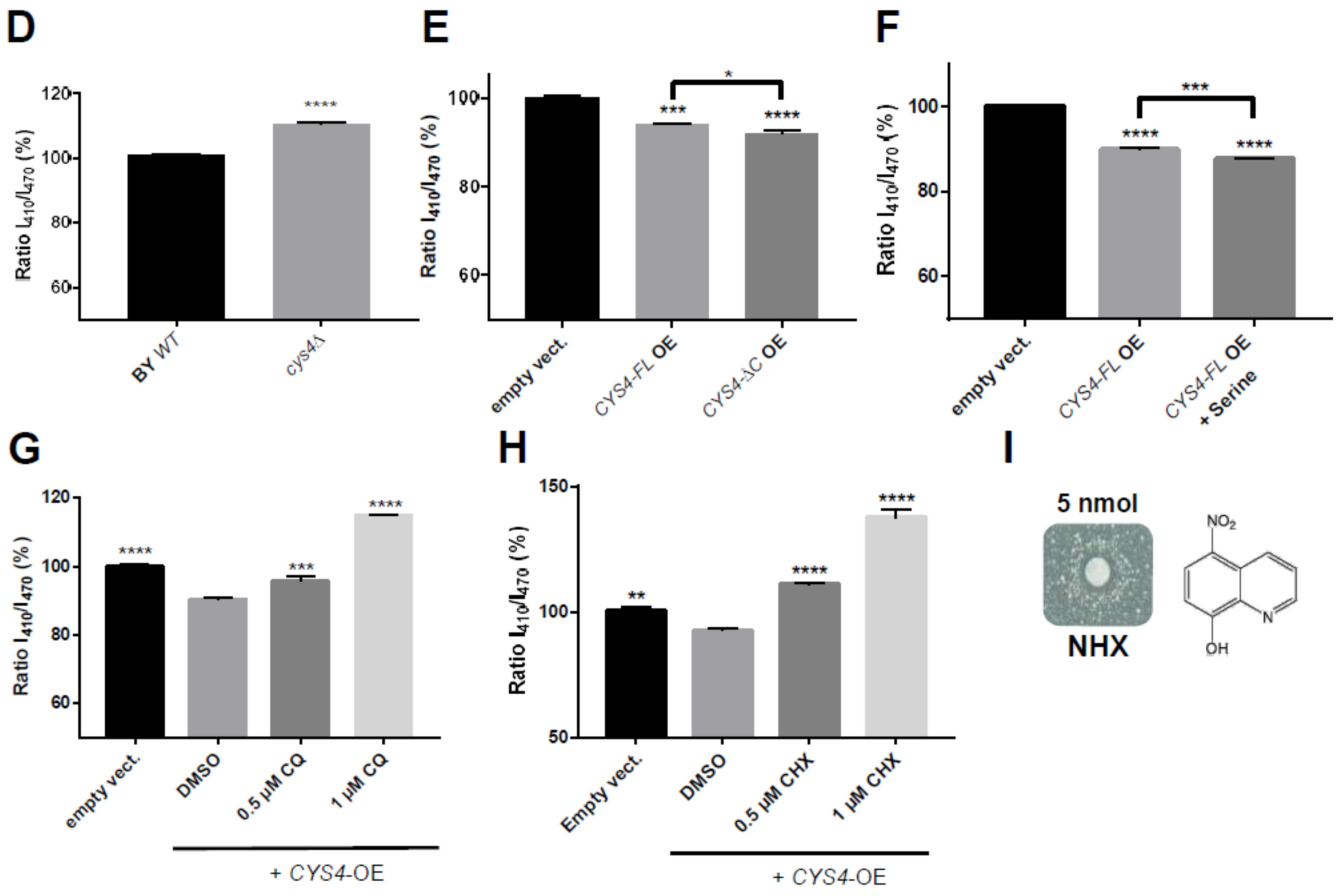
Identification of molecules decreasing the effects of *CYS4* overexpression (*CYS4*-OE) in yeast. (**A**) Simplified representation of the trans-sulphuration pathway. In yeast, *CYS4* encodes the cystathionine beta synthase protein (CBS or Cys4p in yeast), which converts homocysteine and serine into cystathionine. The other enzymes of this pathway are cystathionine gamma-lyase (CSE), (γ-glutamylcysteine synthetase (GCL) and glutathione synthetase (GS). *CYS4* is located at a metabolic hub, its deletion leading to decreased synthesis of cysteine and glutathione in favour of S-adenosylmethionine (SAM), S-adenosylhomocysteine (SAH) and methionine synthesis, whereas *CYS4*-OE favours cysteine and glutathione synthesis at the expense of methionine. (**B**) Methionine auxotrophy of *CYS4*-OE cells. Methionine auxotrophy, revealed by the absence of growth on a methionine-free medium, was assessed by spotting serial dilutions of wildtype yeast cells transformed with two 2µ plasmids either empty or containing full-length (*CYS4*-FL) or a truncated form of *CYS4* (*CYS4*-ΔC), the expression of which is driven by the strong GPD promoter. No serine was added here in order to see the increased methionine auxotrophy obtained with *CYS4*-ΔC compared to *CYS4*-FL. (**C**) Clioquinol (CQ) and chloroxine (CHX) rescue the growth of *CYS4*-OE yeast cells on a methionine-free medium. The indicated amount of drug was added on filters. The positive action of the drugs on cell growth is indicated by the presence of a clear halo surrounding the filters where the molecules were deposited. Due to the gradient effect of the drug deposited on filters, note that these two molecules are toxic at very high concentrations (dark halos around the filters) but are active at lower concentrations (white halos corresponding to the rescue of cell growth). (**D**–**F**) Effect of *CYS4* deletion or OE on cytosolic pH assessed using a pHluorin plasmid. Quantitative measurements of the I_410_/I_470_ ratio of fluorescence showed an increased ratio for *cys4*Δ cells (**D**), indicative of an increased cytosolic pH and a decreased ratio of fluorescence for wildtype cells overexpressing either form of Cys4p (full-length or C-terminal domain deleted) (**E**), indicative of reduced cytosolic pH. Addition of 1.5 mM of serine in the medium of *CYS4*-OE cells also further decreased cytosolic pH (**F**), showing the existence of a direct link between Cys4p enzymatic activity and cytosolic acidification. (**G**,**H**) Dose-dependent rescue of cytosolic acidification of *CYS4*-OE cells by CQ (**G**) and CHX (**H**). Note that at 1 µM, CQ and CHX even increased cytosolic pH above the level of control cells, which is probably the result of their pro-oxidant effect in certain growth conditions, which may partly deplete intracellular glutathione and/or inhibit endogenous Cys4p. (**I**) Nitroxoline (NHX) rescues the growth of *CYS4*-OE yeast cells on a methionine-free medium. Note that NHX was toxic at 10 nmol. (**D**) Student’s *t*-test. (**E**,**F**) One-way ANOVA with Tukey’s post-hoc test. (**G**,**H**): Comparison of each condition with DMSO, one-way ANOVA with Dunnett’s post-hoc test. *, *p* < 0.05; **, *p* < 0.01; ***, *p* < 0.001; ****, *p* < 0.0001.

The CBS (Cystathionine Beta Synthase) gene, located on chromosome 21 in humans, encodes a pyridoxal 5′-phosphate-dependent enzyme catalysing the condensation of homocysteine and serine to form cystathionine. This reaction represents the first committed step in the trans-sulphuration pathway for cysteine and glutathione synthesis (Figure 1A). In mammals, this pathway plays important roles in clearing homocysteine (which is toxic at high levels), in methionine homeostasis and in providing cysteine, the precursor of the major cellular antioxidant glutathione [1]. Increased CBS activity, as observed in Down syndrome (DS) patients, results in decreased plasma levels of homocysteine, methionine, S-adenosylmethionine (SAM) and S-adenosylhomocysteine (SAH), whereas their plasma levels of cystathionine and cysteine are significantly increased [2]. Conversely, decreased CBS activity, as observed in homocystinuria (OMIM 236200) [3], causes increased excretion of homocysteine in patients’ urine and intellectual disability (ID) [4,5]. In DS, the cognitive phenotype has been suggested to relate to abnormal levels of hydrogen sulphide (H_2_S), a major gasotransmitter that is involved in synaptic transmission [6] and which is mainly produced by CBS in the brain [7,8]. In addition, CBS has been reported to be overexpressed in ovarian, breast and colon cancer cells, where it contributes to cancer progression and drug resistance through its role in H_2_S synthesis [9,10,11].

In light of the implication of CBS in the cognitive phenotype of DS [12,13,14] and in cancer [9,10,11], several groups have sought in the last few years to identify inhibitors of CBS using in vitro screening methods, which, unfortunately, only identified compounds with relatively low potency and limited selectivity [15,16,17,18,19], suggesting that in vitro screening based on the recombinant protein is not an efficient approach to identify CBS inhibitors. We consequently developed a new, original screening method based on the overexpression of *CYS4*, the homolog of CBS in the budding yeast *Saccharomyces cerevisiae*, which has the advantage of identifying molecules that interfere with the phenotypical consequences of *CYS4* overexpression without necessarily being direct inhibitors. To ensure the specificity of our screening, we used two different phenotypical readouts to select active and specific molecules. In addition to the methionine auxotrophy that is expected from *CYS4* metabolic function in the trans-sulphuration pathway, we also used the cytosolic acidification phenotype defect that we characterise in this study. Using this screening strategy, we here report the identification of three FDA-approved drugs of the 8-hydroxyquinoline family (clioquinol, chloroxine and nitroxoline) that efficiently decrease CBS activity in different human cellular models. In addition, we provide new findings on the role of intracellular levels of copper and zinc in the regulation of CBS activity, which will help for the development of new therapies for CBS-related human disorders.

## 2. Results

### 2.1. 8-Hydroxyquinoline Derivatives Suppress the Phenotypes Caused by CYS4 Overexpression

As previously shown [13], *CYS4* overexpression (OE) leads to a decreased ability of yeast cells to grow without an external supply of methionine, in agreement with the fact that Cys4p activity consumes methionine through the use of homocysteine (Figure 1A,B). This effect can be enhanced (i) by using a form of Cys4p with its C-terminal auto-inhibitor domain deleted (*CYS4*-ΔC) (Figure 1B and Figure S1A), which has previously been described to have increased enzymatic activity compared to the full-length protein [20], or (ii) by addition into the medium of serine, a substrate of the reaction that increases the stringency of methionine auxotrophy. All this makes this phenotype a convenient readout that can be easily monitored and restored by drugs able to interfere with Cys4p enzymatic activity. Using the methionine auxotrophy phenotype, we screened ≈2200 compounds from chemical libraries consisting mainly of FDA-approved drugs and identified, in addition to disulfiram (DSF) that we previously reported [13], two members of the 8-hydroxyquinoline family, clioquinol (CQ) and chloroxine (CHX), that restored cell growth of *CYS4*-OE cells (Figure 1C). To ensure the specificity of these hits, we sought to identify another phenotype that could be used for secondary screening. Based on the work by Oluwatosin and Kane [21] about a possible link between Cys4p deregulation and defects in vacuolar pH (Figure S1B–D), we observed that *cys4*Δ cells had a more alkaline cytosolic environment than wildtype cells (Figure S2A), which was confirmed by quantitative measurements of the fluorescence ratio obtained from a pHluorin plasmid at 508 nm in response to excitation at two different wavelengths (I410/I470) (Figure 1D) (*p* < 0.0001). Conversely, *CYS4*-OE cells showed increased fluorescence intensity (Figure S2B) and decreased I_410_/I_470_ ratio (Figure 1E) (*p* = 0.0001), suggesting a more acidic cytosolic pH. The fact that *CYS4*-ΔC led to a stronger phenotype than *CYS4*-FL (*p* = 0.0118) suggests that the effect of *CYS4* on cytosolic pH may be linked to its metabolic activity, which was confirmed by the addition of serine in the medium inducing a decrease in cytosolic pH compared to the condition without serine (Figure 1F) (*p* = 0.0003). Finally, experiments using *CYS4* mutants indicated that cytosolic acidification of *CYS4*-OE cells was the consequence of their increased intracellular glutathione levels (Figure S2C–I), confirming that this phenotype is indeed specific of Cys4p enzymatic function. It is important to note that, although these cytosolic pH variations seemed to be of low amplitude, this is to be expected knowing all the consequences to cellular functions that can occur from changes to intracellular pH. We found that the results were reproducible and robust enough to be used as a specific readout of Cys4p metabolic function in a secondary screening step to confirm the specificity of the drugs or the genetic suppressors identified. We thus tested the effects of CQ and CHX on cytosolic pH and showed that these two molecules, in addition to being able to rescue methionine auxotrophy, also reduced the cytosolic acidification of *CYS4*-OE cells (Figure 1G,H). Then, in order to study a few other 8-hydroxyquinoline are FDA-approved drugs, we tested other derivatives and further showed that nitroxoline (NHX) was able to restore cell growth of yeast cells on a medium without methionine (Figure 1I).

### 2.2. CQ, CHX and NHX Reduce Human CBS Enzymatic Activity in Liver Cells

**Figure 2:**
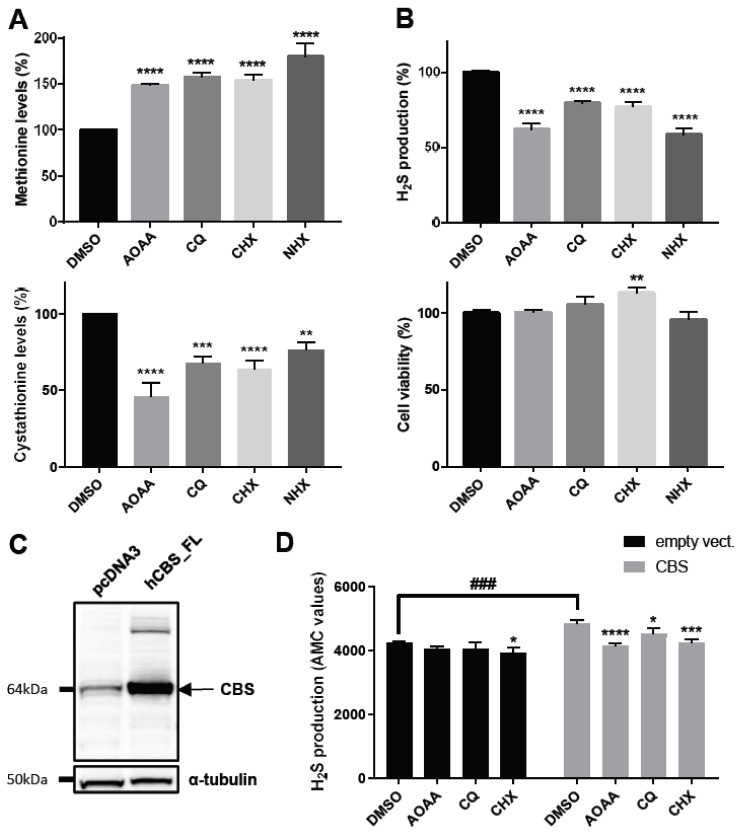
Molecules reducing the phenotypes induced by *CYS4*-OE-induced a decrease in human CBS activity. (**A**) Effects of CQ, CHX and NHX on the intracellular levels of methionine (upper panel) and cystathionine (lower panel). A 24 h treatment of HepG2 cells with 500 µM of AOAA, used as a positive control, and 15 µM of CQ, CHX or NHX resulted in decreased intracellular levels of methionine and increased levels of cystathionine compared to cells treated with the vehicle (DMSO). (**B**) Effects of CQ, CHX and NHX on H_2_S production in HepG2 cells. H_2_S production and cell viability were assessed by AzMC probe and WST-8 assay, respectively. A 24 h treatment of HepG2 cells with 500 µM of AOAA and 20 µM of CQ, CHX or NHX resulted in decreased H_2_S production (upper panel) without decreasing cell viability (lower panel). Note that CHX tended to increase cell proliferation at 20 µM (see also Figure S3). (**C**) Western blot showing the level of expression of CBS in cellular lysates of HepG2 cells transfected with the expression plasmid pcDNA3-hCBS compared to cells transfected with the pcDNA3 empty vector. (**D**) Measure of H_2_S production in cell lysates obtained from pcDNA3 (empty vector) or CBS-transfected cells. Each molecule was incubated at 50 µM with the indicated cell lysate for 2 h, and the level of H_2_S production was assessed by AzMC probe. Note that in CBS-transfected cells, AOAA decreases the level of H_2_S production to that obtained in control cells. Student’s *t*-test: ###, *p* < 0.001. (**A**,**B**,**D**) Comparison of each condition with DMSO, one-way ANOVA with Dunnett’s post-hoc test: *, *p* < 0.05; **, *p* < 0.01; ***, *p* < 0.001, ****, *p* < 0.0001.

To validate the activity of CQ, CHX and NHX on CBS enzymatic activity, we used the human hepatoma HepG2 cell line because CBS is known to be highly expressed and active in the liver. As CBS triplication leads to decreased levels of methionine and increased levels of cystathionine in the serum of DS patients [2], we first measured by mass spectrometry the intracellular levels of these amino acids following a 24 h treatment with 15 µM of CQ, CHX or NHX. As a positive control, we used aminooxyacetic acid (AOAA, at 500 µM), which has been shown to inhibit multiple PLP-dependent enzymes, including CBS [22]. It is, however, important to note that AOAA has limited selectivity, as it also inhibits several oxidoreductases, transferases, hydrolases and isomerases [23]. As shown in Figure 2A, CQ, CHX and NHX were all able to significantly increase the intracellular level of methionine (upper panel) by 57%, 54% and 80%, respectively, whereas the level of cystathionine (lower panel) was decreased by 32%, 36%, and 24%, respectively, confirming that similarly to AOAA (which increased methionine level by 48% and decreased cystathionine level by 54%), CQ, CHX and NHX could reduce CBS enzymatic activity. In parallel, as CBS is also involved in hydrogen sulphide (H_2_S) production, we measured its levels using the 7-Azido-4-methylcoumarin (AzMC) fluorogenic probe. A 24 h treatment with 20 µM of either CQ, CHX or NHX significantly decreased H_2_S production levels (upper panel) by 21%, 23% and 42%, respectively, compared to 38% for 500 µM AOAA, without decreasing cell viability (Figure 2B, lower panel). Furthermore, this effect was observed in a dose-dependent manner up to 50µM, a concentration for which CQ and CHX, respectively, reached 60% and 55% decreased H_2_S production, whereas NHX reached a maximum of 45–50% reduction of H_2_S production at concentrations above 20 µM (Figure S3A–C).

Then, to rule out a possible effect of the compounds on the regulation of CBS expression levels and/or protein stability, we tested the ability of CQ and CHX to reduce CBS activity using cellular lysates obtained from HepG2 cells transfected either with the pcDNA3 vector or with human CBS cDNA (Figure 2C). As shown in Figure 2D, CBS-transfected cells showed significantly increased H_2_S production. As expected, AOAA decreased H_2_S production more strongly in CBS-transfected cells (by 14.2%, *p* = 0.0001) compared to control cells (4.4%, *p* = 0.2553, NS). Similarly, CHX, and to a lesser extent CQ, were also able to decrease H_2_S production, more particularly in lysates obtained from CBS-transfected cells (6.6% in CBS-transfected cells versus 4.9% in control cells for CQ and 12.1% versus 5.7% for CHX) (Figure 2D). These results confirm that these two molecules, similarly to AOAA, most likely target CBS enzymatic activity but not the regulation of its expression level or its protein stability. Unfortunately, we were not able to test NHX using this assay, as this compound was found to non-specifically interact with produced H_2_S (probably through its nitro group, which is not present in CQ and CHX) (data not shown). Taken together, these data confirm the ability of CQ, CHX and NHX to specifically decrease CBS activity in human cells.

### 2.3. Decreasing Intracellular Copper Levels Reduces Cystathionine Beta Synthase Enzymatic Activity

Both CHX and CQ are clinically employed as antimicrobial agents to treat a wide range of intestinal diseases. Similarly, NHX is used to treat urinary tract infections [24]. In the last years, CQ has also been proposed as a therapeutic drug for the treatment of Alzheimer’s [25] and Huntington’s diseases [26] based on its properties of metal homeostasis modulation [27,28]. CQ, CHX and NHX are bidentate chelators that bind metal ions through the oxygen of their hydroxyl group and the nitrogen of their quinoline, with a preference for Cu(II) and Zn(II) [29]. To determine whether this property is involved in the capacity of these three molecules to reduce CBS enzymatic activity, we tested several structural derivatives of CQ, CHX or NHX, both in yeast using methionine auxotrophy and in HepG2 cells using H_2_S production levels as readouts (Table S4). CQ, CHX and NHX are based on an 8-hydroxyquinoline scaffold, and both nitrogen and oxygen atoms are crucial for the activity, as confirmed by the complete loss of activity of compounds **9** and **10** (Table S4). Moreover, the presence of electron withdrawing groups, such as halogens (compounds **2**–**7**, Table S4) or nitro (compound **1**, Table S4) at positions 5 and 7, improves CBS activity inhibition, whereas electron donating group NH_2_ at position 5 (compound **11**) led to an inactive compound. This structure–activity–relationship (SAR) study is consistent with the hypothesis that the inhibition of CBS activity by 8-hydroxyquinoline depends on their ability to bind metal ions through the molecular pincer formed by both oxygen and nitrogen atoms.

**Figure 3:**
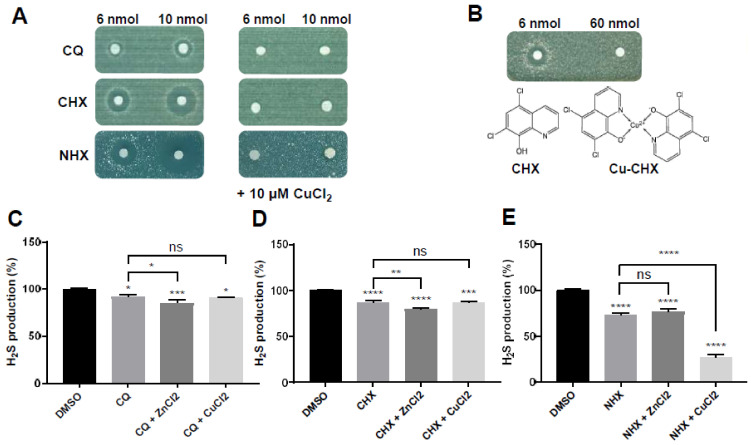

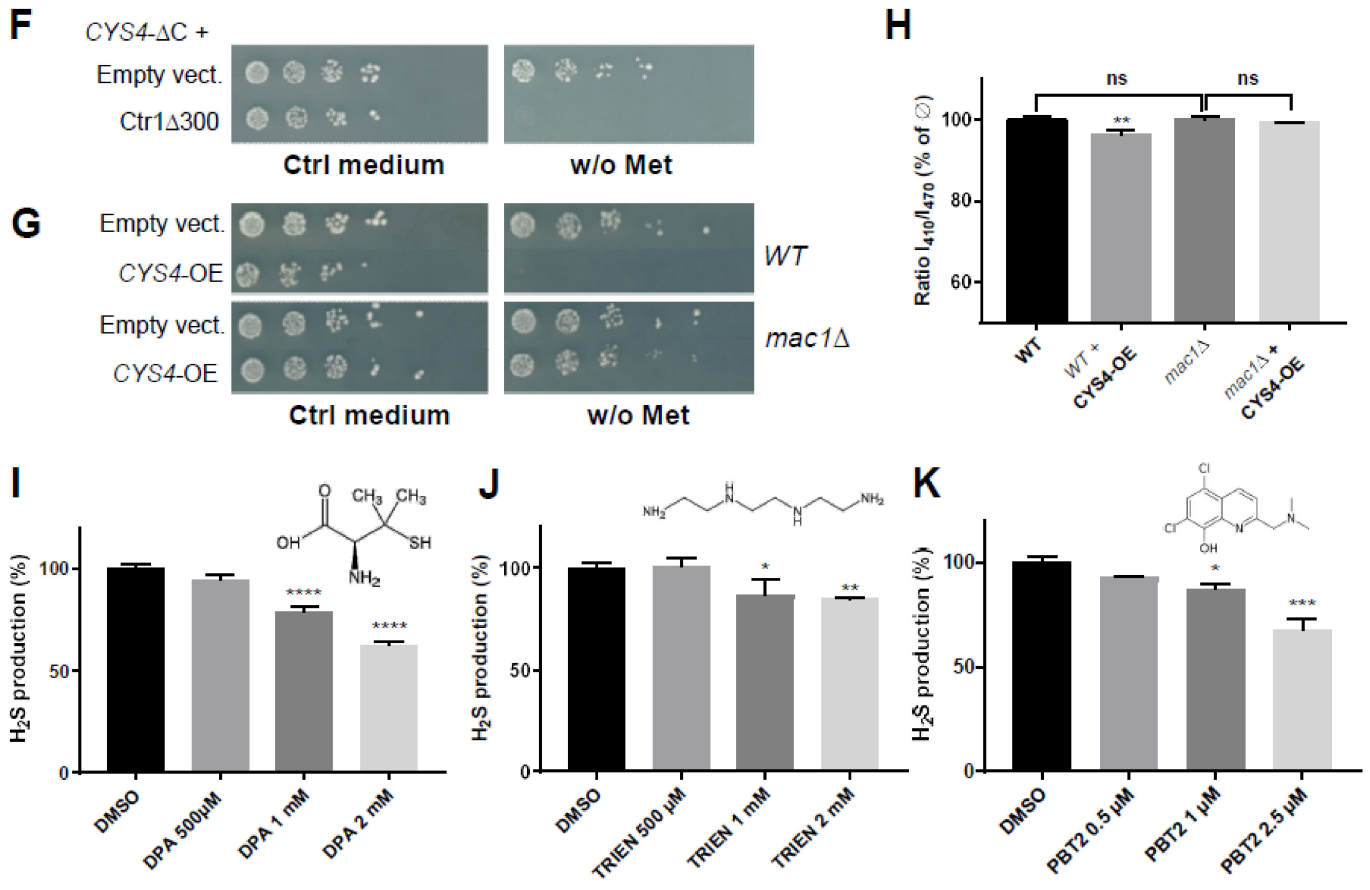
Decreasing intracellular copper levels reduces cystathionine beta synthase enzymatic activity. (**A**) Effect of copper enrichment in the medium. *CYS4*-OE cells were grown on a methionine-free medium containing 1.5 mM of serine (**left panel**). Addition of 10 µM of CuCl_2_ in the medium (**right panel**) completely abolished the effect of CQ, CHX or NHX on growth restoration of *CYS4*-OE cells. Note that the cellular toxicity of the drugs at high doses (indicated by dark halos around the filters) was also completely abolished by CuCl_2_. (**B**) Test of CHX complexed to copper. A CHX-copper complex (filter on the right side of the plate) was unable to restore cell growth of *CYS4*-OE cells on a free-methionine medium, in contrast to CHX alone (filter on the left). (**C**–**E**) Effect of a 24 h incubation of HepG2 cells with a combination of the drug with zinc salts or copper salts on H_2_S production. Addition of 2.5 µM of CuCl_2_ did not have any significant effect on the action of 10 µM of CQ (**C**) or CHX (**D**) but significantly increased the toxicity of NHX ((**E**) and Figure S4C–E). The addition of 10 µM of ZnCl_2_ slightly increased CQ (**C**) or CHX (**D**) activity but did not have any effect on NHX (**E**). Higher concentrations of ZnCl_2_ or CuCl_2_ combined with CQ, CHX or NHX decreased cell viability (data not shown). (**F**) Effect of intracellular copper enrichment on the methionine auxotrophy phenotype of *CYS4*-OE cells. Expression of *Ctr1*Δ300 (leading to increased intracellular copper levels) exacerbated methionine auxotrophy. This assay was performed in a yeast strain transfected with only one vector expressing *CYS4*-ΔC instead of two in order to have an intermediate stringency of methionine auxotrophy due to *CYS4*-OE. Strains expressing *Ctr1*Δ300 or control empty plasmids were spotted in serial dilutions on control medium containing methionine (**left panel**) and on a methionine-free medium (**right panel**) to assess their growth. (**G**,**H**) Effect of intracellular copper depletion on cellular phenotypes of *CYS4*-OE cells. (**G**) *MAC1* encodes a transcription factor activating the expression of copper transporters in yeast, and its deletion has been shown to induce intracellular copper depletion. In the absence of *MAC1*, *CYS4*-*OE* did not induce methionine auxotrophy. (**H**) Similarly, *CYS4*-OE was not able to induce cytosolic acidification in a *Mac1*Δ strain. (**I**–**K**) Effect of copper chelators on H_2_S production in HepG2 cells. A 24 h incubation with three copper chelators, D-penicillamine (**I**), trientine (**J**) and PBT2 (**K**), resulted in decreased H_2_S production in HepG2 cells. Cell viability was assessed by WST-8 assay (Figure S4F–H). (**C**–**E**,**H**) One-way ANOVA with Tukey’s post-hoc test. (**I**–**K**) Comparison of each condition with DMSO, one-way ANOVA with Dunnett’s post-hoc test. ns: not statistically significant; *, *p* < 0.05; **, *p* < 0.01; ***, *p* < 0.001, ****, *p* < 0.0001.

As copper was previously reported to regulate the activity of recombinant CBS in vitro [30], we first evaluated the effects of CuSO_4_ or CuCl_2_ on the capacity of CQ, CHX and NHX to reduce both methionine auxotrophy in yeast and H_2_S production in HepG2 cells. Copper salts on their own were not able to rescue CYS4 OE cell growth on a methionine-free medium (Figure S4A) and had no effect on H_2_S production in HepG2 cells (Figure S4B). However, addition of copper salts to the filters (data not shown) or into the medium (Figure 3A) completely abolished the capacity of CQ, CHX and NHX to restore CYS4-OE cell growth on medium without methionine. Similarly, CHX complexed to copper completely lost the ability to restore cell growth (Figure 3B), suggesting that CHX has to be either “free” or bound to a metal other than copper to have a positive effect on CYS4-OE cell growth. Regarding H_2_S production in HepG2 cells, addition of copper salts together with free CHX or CQ did not significantly affect the H_2_S decrease induced by both hydroxyquinoline derivatives (Figure 3C,D and Figure S4C,D), suggesting that these two compounds may have a rather low affinity for extracellular copper. On the other hand, copper salts added to NHX drastically decreased H_2_S production, but this effect was mainly due to cell toxicity (Figure 3E and Figure S4E), suggesting that NHX may act here as a copper ionophore and that bringing too much copper into the cell is detrimental for cell viability. Unfortunately, we could not assess H_2_S production using cellular lysates as, similar to what has been reported in several studies [19,31], we observed that copper ions reacted either with the newly produced H_2_S and/or with the AzMC probe, inactivating the AMC fluorescence reaction at concentrations of CuCl_2_ as low as 1 µM.

We further investigated the impact of enrichment or depletion of intracellular levels of copper using genetic tools in yeast cells. We first expressed a constitutively active form of the copper transporter Ctr1p (Ctr1Δ300), which led to a two-fold increase in intracellular copper levels [32]. As shown in Figure 3F, Ctr1Δ300 expression exacerbated methionine auxotrophy of *CYS4*-OE cells. We then used a strain with *MAC1* deleted; *MAC1* encodes the transcription factor activating the expression of copper transporters in yeast. *MAC1* deletion, which leads to a reduction in the levels of intracellular copper [33], alleviated methionine auxotrophy (Figure 3G) as well as cytosolic acidification (Figure 3H) of *CYS4*-OE cells. Taken together, these results suggest that Cys4p activity is dependent on copper, as decreasing intracellular copper levels reduces its activity. To further test this hypothesis, we used D-penicillamine (DPA) and trientine (TRIEN), two drugs used to chelate excess copper in patients with Wilson’s disease. As shown in Figure 3I,J, high concentrations of these two copper chelators significantly decreased H_2_S production, up to 38% and 15%, respectively, although 2 mM DPA also affected the viability of HepG2 cells (Figure S4F,G). We also tested PBT2 (2-(dimethylamino)methyl-5,7-dichloro-8-hydroxyquinoline), an 8-hydroxyquinoline derivative bearing a second nitrogen atom to reinforce its metal binding potential, which has been developed for the treatment of both Alzheimer’s and Huntington’s diseases, and which restores metal ion homeostasis in the brain [34,35]. As shown in Figure 3K, PBT2 was also able to decrease H_2_S production, but at much lower concentrations than DPA and TRIEN (1µM versus 1 mM, respectively), reaching an H_2_S production decrease of 32% at 2.5 µM without reducing cell viability (Figure S4H). Altogether, these data confirm that decreasing intracellular copper levels reduces CBS activity.

### 2.4. Zinc Ionophores Reduce the Effects of Cystathionine Beta Synthase Overexpression

**Figure 4:**
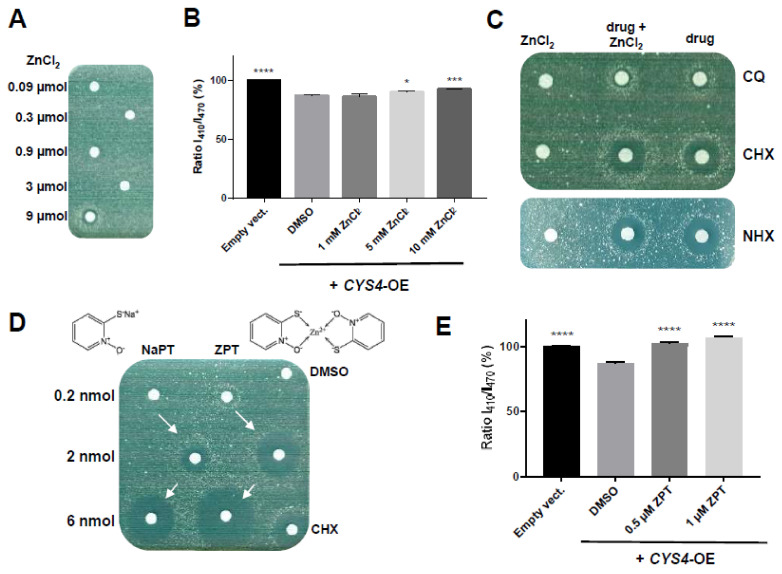

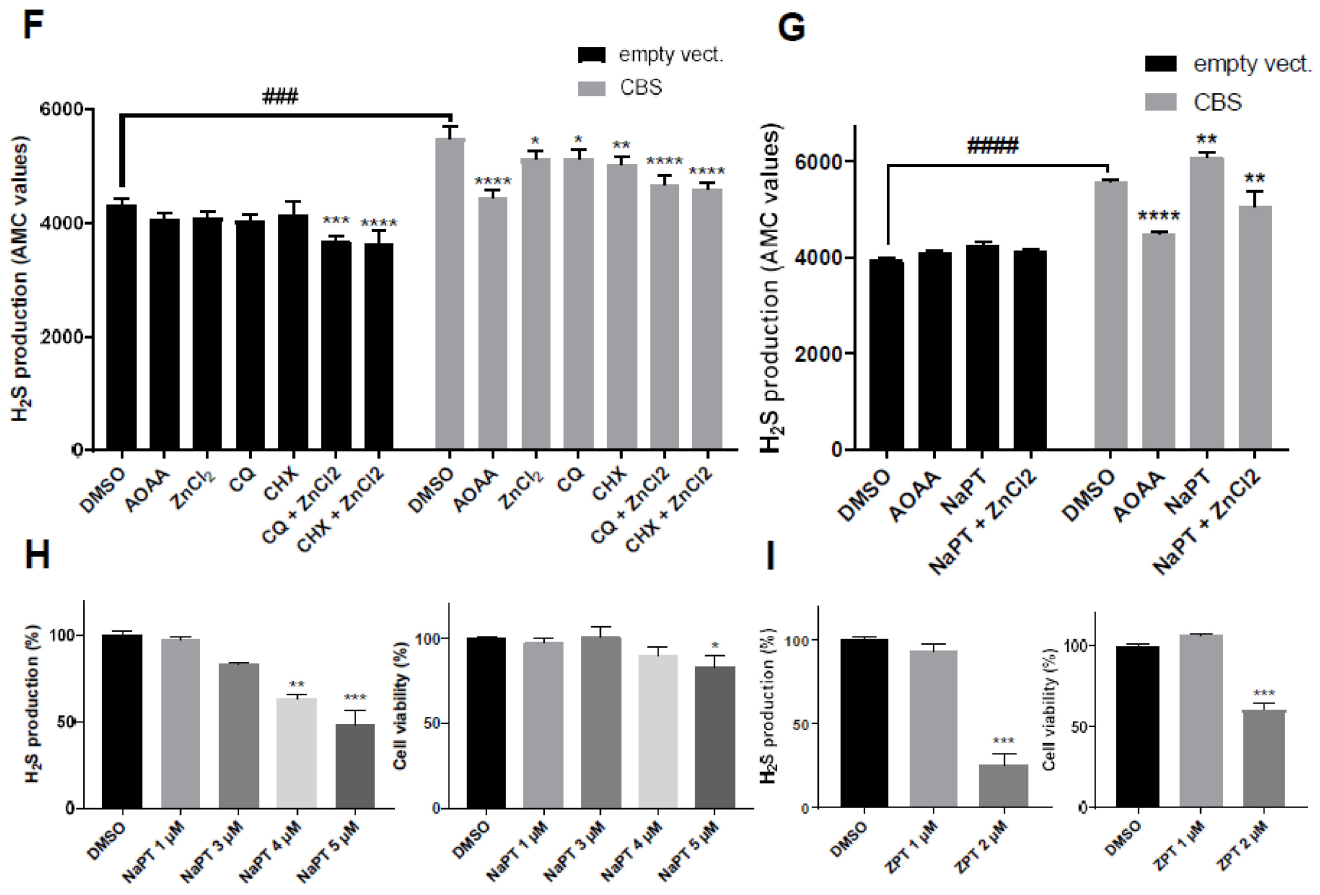
Effect of zinc enrichment on cystathionine beta synthase activity. (**A**–**E**) Effect of zinc enrichment of the medium on the phenotypes of *CYS*4-OE cells. (**A**) *CYS4*-OE cells were grown on a methionine-free medium, and increasing amounts of ZnCl_2_ were loaded on filters. At the highest dose tested (9 µmol), ZnCl_2_ on its own was sufficient to restore cell growth of *CYS4*-OE cells on a methionine-free medium. (**B**) Addition of increasing amounts of ZnCl_2_ increased the intracellular pH of *CYS4*-OE cells in a dose-dependent manner. (**C**) *CYS4*-OE cells were grown on a methionine-free medium, and 0.3 µmol of ZnCl_2_ was added either alone (left filter) or in combination with the tested drug (middle filter). Compared to the drug alone (right filter; 6 nmol of CQ, CHX or NHX), the addition of ZnCl_2_ slightly increased the activity of CQ and CHX, as shown by their increased toxicity and the decreased concentrations of the molecule necessary to restore cell growth on a methionine-free medium (indicated by the larger distance of the halo from the filter). However, ZnCl_2_ did not appear to have any obvious effect on NHX action, which is consistent with published findings that NHX is not a zinc ionophore. (**D**) Pyrithione, a zinc ionophore, was able to rescue cell growth of *CYS4*-OE cells on a methionine-free medium. Note that zinc pyrithione (ZPT) was more active and more toxic compared to the same amounts of sodium pyrithione (NaPT). (**E**) Similarly, zinc pyrithione also increased the cytosolic pH of *CYS4*-OE cells. (**F**) Effect of the addition of ZnCl_2_ to CHX and CQ on H_2_S production in cell lysates. The effect of 50 µM of CQ or CHX on H_2_S production was visible after a 2 h incubation with cell lysates obtained from pcDNA3- or CBS-transfected HepG2 cells. The efficiency of both CQ and CHX was more pronounced in CBS-transfected cells, which produced higher levels of H_2_S compared to control-transfected cells. The action of CQ and CHX was also enhanced by 50 µM of ZnCl_2_ in cell lysates. AOAA (50 µM) was used as a positive control. (**G**) Effect of the addition of ZnCl_2_ on NaPT in cell lysates. Whereas the addition of 50 µM of NaPT on its own increased the production of H_2_S, the addition of 50 µM of ZnCl_2_ to 50 µM of NaPT significantly decreased the production of H_2_S after a 2 h incubation with cell lysates obtained from CBS-transfected HepG2 cells. (**H**,**I**) Dose-dependent effect of pyrithione complexed to sodium (NaPT) or to zinc (ZPT) on H_2_S production after a 24 h incubation of HepG2 cells. Cell viability (**right panel**) was assessed by WST-8 assay. (**B**,**E**–**I**) Comparison of each condition with DMSO, one-way ANOVA with Dunnett’s post-hoc test: *, *p* < 0.05; **, *p* < 0.01; ***, *p* < 0.001, ****, *p* < 0.0001. (**F**,**G**) Student’s *t*-test: ###, *p* < 0.001, ####, *p* < 0.0001.

Based on the high membrane permeability and zinc-binding property of CQ and CHX, a few studies have suggested a role for both compounds as zinc ionophores [36,37,38,39], shuttling free zinc in or out of cells, depending on the free zinc concentration gradient. We thus also assessed the consequences of the modulation of intracellular zinc levels on CBS activity. ZnCl_2_ on its own was able, at the highest dose deposited on filters (9 µmol), to restore cell growth of *CYS4*-OE cells on a methionine-free medium (Figure 4A). Similarly, ZnCl_2_ was able to increase the intracellular pH of *CYS4*-OE yeast cells (Figure 4B) (*p* = 0.0008 at 10 mM). In addition, ZnCl_2_ plus CQ, CHX or NHX on filters appeared to enhance the effect of both CQ and CHX, as shown by the increased thickness of their halo of growth restoration (Figure 4C). No effect of ZnCl_2_ on NHX was visible, which is in agreement with previous reports showing that, unlike CQ and CHX, NHX is not a zinc ionophore [40]. Then, we tested the zinc ionophore pyrithione [29], and its ability to rescue *CYS4*-OE cell growth was more efficient when complexed with zinc (ZPT) than complexed to sodium (NaPT) (Figure 4D). ZPT was also more toxic, suggesting that bringing in too much zinc can be detrimental to the cells. As expected, ZPT was also able to increase the intracellular pH of *CYS4*-OE yeast cells (Figure 4E) (*p* < 0.0001 at 0.5 µM). Taken together, these results show that increasing intracellular zinc levels contributes to the rescue of *CYS4*-OE induced phenotypes. Concerning H_2_S production, zinc salts on their own had no effect on H_2_S production in HepG2 cells (Figure S4I,J). However, addition of zinc salts appeared to slightly increase CQ and CHX capacity to reduce H_2_S in live HepG2 cells (Figure 3C,D) (respectively, *p* = 0.348 and *p* = 0.040) but did not have any significant effect on NHX (Figure 3E and Figure S4C–E). Similarly, the action of CQ and CHX was significantly enhanced by zinc salts when H_2_S production was measured in cell lysates, both in CBS-transfected cells and control cells (Figure 4F). Then, we assessed the effect of the zinc ionophore pyrithione in both HepG2 cell lysates (Figure 4G) and in live cells (Figure 4H,I). In cellular lysates, NaPT was able to decrease H_2_S production only in the presence of ZnCl_2_ and only in lysates from CBS-transfected cells (Figure 4G). In live cells, NaPT showed a dose-dependent decrease in H_2_S production, reaching ≈50% at 5 µM (Figure 4H), while ZPT was active at a concentration as low as 2 µM but also impacted cell viability (Figure 4I). Taken together, these data suggest that, in addition to copper chelation, increasing intracellular levels of zinc participates in the reduction of CBS activity.

### 2.5. Effect of CQ, CHX and NHX on CBS Activity Inhibition in Other Human Cellular Models

**Figure 5:**
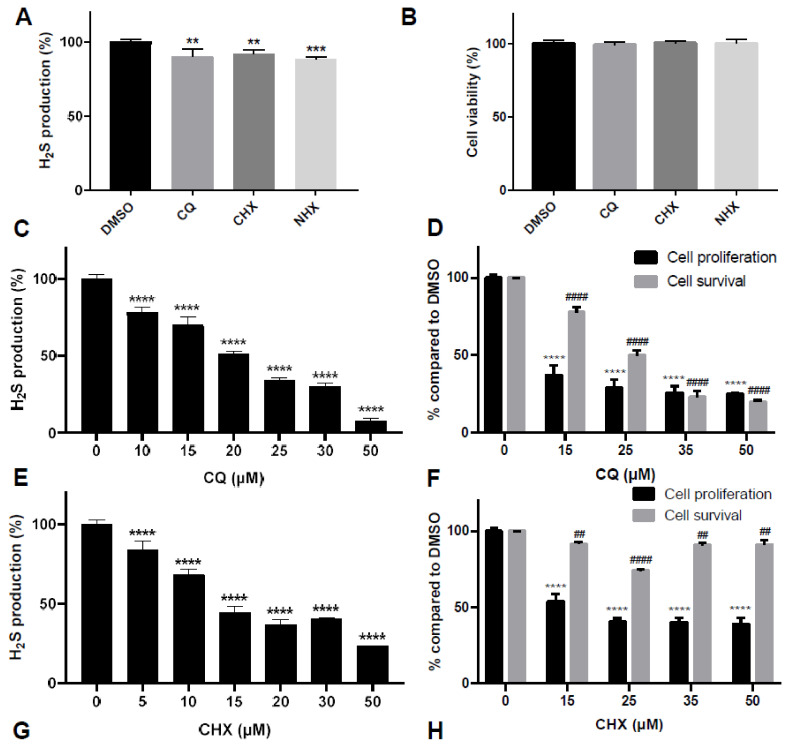

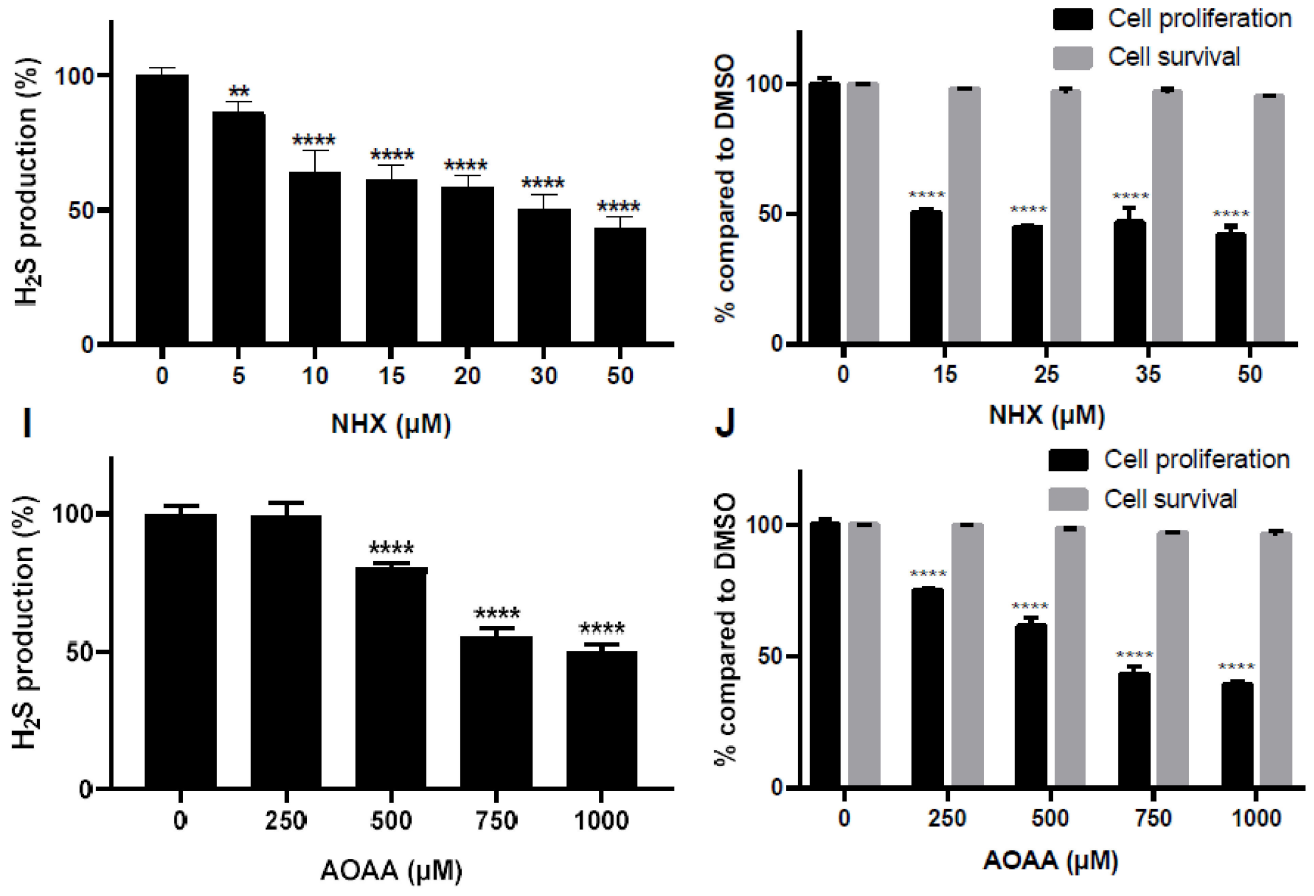
Effect of CQ, CHX and NHX in pathophysiological models of CBS overexpression. (**A**,**B**) Reduction of H_2_S production by CQ, CHX or NHX in fibroblasts of an adult DS patient. A 24 h treatment of fibroblasts obtained from an adult DS patient with 15 µM of CQ, CHX or NHX showed a significant decrease in H_2_S production (**A**) without affecting cell viability (**B**). This reduction in H_2_S production is modest and was not improved much by higher drug concentrations, but this is probably due to the fact that fibroblasts produce less H_2_S compared to HepG2 cells (~7–10 times less based on our AzMC measurements). (**C**–**J**) Effect of CQ, CHX, NHX and AOAA on H_2_S production, cell proliferation and cell survival in HCT116 cells. A 24 h treatment of HCT116 cells with 10–50 µM of CQ (**A**,**B**) or CHX (**C**,**D**) or NHX (**E**,**F**) induced a dose-dependent reduction in H_2_S production (**A**,**C**,**E**) and decreased cell proliferation (**B**,**D**,**F**); it also decreased cell survival for CQ and CHX (**B**,**D**). AOAA, used as a control, and NHX similarly decreased H_2_S production and cell proliferation, but AOAA was used at a much higher concentration than NHX (**G**,**H**). Comparison of each condition with DMSO, one-way ANOVA with Dunnett’s post-hoc test: ** or ^##^, *p* < 0.01; ***, *p* < 0.001, **** or ^####^, *p* < 0.0001.

As it has recently been suggested that the deregulation of H_2_S production by CBS triplication may contribute to the cognitive phenotype of DS patients [12,14], we then thought to assess the effect of CQ, CHX and NHX on H_2_S production in fibroblasts from adult DS patients. As shown in Figure 5A,B, a 24 h treatment of 15µM of CQ, CHX and NHX significantly decreased H_2_S production by ≈10% in DS fibroblasts without affecting cell viability. This effect may appear rather modest, but although this cell type is one of the easiest to obtain from patients, it is probably not the most adequate for the study of CBS activity, as both CBS expression and H_2_S production are low in this cell type and have been found to be decreased in replicative senescent adult human dermal fibroblasts [41].

Then, as CBS overexpression has also been shown to stimulate cell proliferation in HCT116 colon cancer, and therapeutic manipulation of H_2_S has recently been proposed as an anticancer candidate [9,42,43], we assessed whether CQ, CHX and NHX may be able to decrease H_2_S production and consequently cell proliferation in HCT116 cells. As shown in Figure 5C–H, we observed that, indeed, 24 h treatments with CQ, CHX and NHX, used at concentrations that are not toxic and that do not affect proliferation of HepG2 cells (Figure S3A–C), were able to efficiently decrease both H_2_S production and cell proliferation (Figure 5C–H). CQ, and to a lesser extent CHX, appeared to be the most efficient molecule to reduce H_2_S production and cell proliferation in HCT116 after 24 h of treatment (Figure 5C,D). However, both drugs also had a significant impact on cell viability, especially CQ (Figure 5D). NHX gave very similar results to AOAA, although at 20 times lower concentration (Figure 5G–J). NHX decreased H_2_S production up to 57% at 50 µM (versus 50% at 1 mM for AOAA) and cell proliferation up to 58% (versus 61% at 1 mM for AOAA). Taken together, these data show that the three compounds identified in this study are able to decrease HCT116 cell proliferation by reducing CBS-derived H_2_S production. Although CQ, and to a lesser extent CHX, also impacted cell viability, NHX gave very similar results to AOAA but at much lower concentrations, showing that NHX is a much more potent inhibitor of CBS enzymatic activity.

## 3. Discussion

### 3.1. Identification of 8-Hydroxyquinoline Derivatives That Decrease Cystathionine Beta Synthase Enzymatic Activity

We here describe the identification of the 8-hydroxyquinolines CQ, CHX and NHX as FDA-approved drugs that efficiently reduced the effects of *CYS4* OE in yeast. In addition, these three molecules decreased H_2_S production and restored amino acid levels in the trans-sulphuration pathway in HepG2 cells at much lower concentrations than AOAA (10–20 µM for CQ, CHX and NHX versus 0.5–1 mM for AOAA), the only previously described molecule able to inhibit CBS activity in cellular models [23].

These results validate our screening method as an effective approach to identify compounds regulating CBS enzymatic activity using molecular mechanisms conserved in yeast and humans. One major advantage of this approach is that the compounds were identified using a phenotypic model combining both genetic and chemical biology aspects, which allows the identification of molecules that interfere with the phenotypical consequences of *CYS4* OE without necessarily being direct inhibitors. Interestingly, we isolated very few molecules (only 3 out of ~2200 molecules tested, corresponding to <0.2%) in this drug screening, indicative of a high selectivity.

### 3.2. Hypotheses on the Mode of Action of CQ, CHX and NHX

CQ and CHX were found to be able to reduce H_2_S production in cellular lysates, ruling out a possible effect on the regulation of CBS expression levels and/or protein stability. The fact that CQ and CHX had a significantly more pronounced effect on H_2_S production in lysates obtained from CBS-transfected HepG2 cells than in control cells (Figure 2F) suggests that their action targets CBS-mediated enzymatic reactions. However, it seems rather unlikely that these molecules are direct inhibitors of CBS. Indeed, Kashiwamata and Greenberg previously tested 8-hydroxyquinoline (compound **8**, Table S4) and found that 1 mM had no effect on the activity of purified CBS enzyme [44].

Our SAR study on the 8-hydroxyquinoline scaffold is consistent with the hypothesis that the observed decrease of CBS activity depends on the ability of 8-hydroxyquinolines to bind metal ions (Table S4). It is thus possible that by regulating intracellular levels of copper and zinc ions and/or their bioavailability, CQ and CHX may positively or negatively act on other enzymes. For example, both methionine synthase (which is responsible for the regeneration of methionine from homocysteine) and betaine–homocysteine S-methyltransferase (BHMT, a key liver enzyme that is important for homocysteine homeostasis and accounts for approximately half of the methionine synthesized in the liver) are zinc-dependent enzymes [45,46], and it is thus possible that increasing zinc bioavailability can activate one or both of these two enzymes, increasing the production of methionine at the expense of homocysteine and cysteine, the main substrates of CBS. This hypothesis is in agreement with the effect of zinc ionophores in our experiments. However, as NHX is not supposed to have zinc ionophore properties [40], it is probably not the only mode of action of the three compounds we identified in this study.

Another possible mode of action of CQ, CHX and NHX is through copper chelation and/or redistribution. Indeed, mutual antagonism between copper and zinc has often been described [47], and copper itself has been previously suggested to directly inhibit CBS [30]. However, these data were generated in vitro and, as copper ions have a strong affinity for thiols, it is likely that in vitro, copper ions physically interact with key cysteine residues of CBS, thus impeding its enzymatic activity [48]. In an in vivo context, accessible copper ions are probably not present in a sufficient amount to be able to directly interact with the active site of CBS. Accordingly, increasing amounts of copper were not able to decrease CBS activity in HepG2 cells, and there was even a trend towards increased activity, although it did not reach statistical significance (Figure S4B). In yeast, increased intracellular copper levels have been shown to lead to the activation of the transsulfuration pathway [32], probably in order to produce more GSH and cysteine, which are copper scavengers. On the contrary, Tamura and colleagues have shown that rats fed a low-copper diet showed higher plasma homocysteine concentrations compared to the control group, suggesting decreased activity of the transsulfuration pathway [49]. In addition, decreased cellular levels of copper are usually associated with decreased functioning of certain proteins, such as the superoxide dismutase SOD1. It is thus possible that through copper chelation, CQ, CHX and NHX decrease ROS levels, which may in turn cause the reduction of CBS activity. These data are in line with previous observations showing that CBS activity is strongly dependent on cellular redox state [1,50,51]. Accordingly, our data confirm that all the molecules identified in this study have antioxidant properties at concentrations that decrease H_2_S production (Figure S5). None of the other metallic cations that we tested (Fe(II), Mn(II) and Mg(II)) had any effect except for iron, which mimicked copper’s effects, although to a lesser extent (data not shown). These observations are in agreement with the facts that CQ also has an affinity for iron [52] and that iron also has several oxidation states, similar to copper, and can interfere with oxidative stress. More work is currently ongoing to test these hypotheses.

### 3.3. CQ, CHX and NHX Efficiently Decrease CBS Activity in Other Cellular Models

Although two other enzymes are also involved in H_2_S production, cystathionine γ-lyase (CSE) and 3 mercaptopyruvate sulfurtransferase (3-MST), several studies have shown that decreasing H_2_S production by specifically targeting CBS expression in HCT116 cells significantly reduces cell proliferation [9]. We here confirmed the inhibitory effects of CQ, CHX and NHX on CBS in HCT116 cells. These three compounds efficiently decreased H_2_S production in a dose-dependent manner in HCT116 cells and subsequently decreased cell proliferation after 24 h of treatment. In addition, CQ, CHX and NHX were much more efficient than AOAA in decreasing HCT116 cell proliferation and cell viability. More studies will be necessary to evaluate the possible use of these three drugs in CBS-overexpressing cancer models, but it is encouraging to note that both CQ and NHX have previously been reported to successfully reduce the viability of different human cancer cell lines [37,40].

The use of CBS inhibitors has recently been suggested as a potential therapeutic option to reduce intellectual disability severity in DS patients [53]. In our experiments, we detected a moderate but significant effect of CQ, CHX and NHX on H_2_S production in fibroblasts. However, this cell type is not the best one to study the impact of CBS expression on H_2_S production. The use of cerebral organoids derived from DS patients’ induced pluripotent stem cells (iPSC) should provide more reliable information about the effects of CBS inhibitor candidates on neuronal cells. Nevertheless, the study of CQ, CHX and NHX in DS may be worth pursuing, especially as CQ and PBT2 have shown neuroprotective properties in Alzheimer’s disease [39,54], which affects ≈1/3 of DS patients in their fifties. Similarly, zinc metabolism has been reported to be impaired in patients with DS [55], possibly because of the triplication of *SOD1* (also located on chromosome 21). Zinc supplementation in DS patients has been shown to be beneficial for several aspects, including immune and endocrinological problems associated with thyroid dysregulation. It would thus be interesting to see whether zinc combined with ionophores such as CQ may also help at the cognitive level.

In conclusion, we here described a new method for drug or genetic screening based on two different yeast phenotypes that are specific to CBS overexpression. In addition to methionine auxotrophy, we here described a new effect of CBS level modulation on cytosolic pH. Thanks to these two specific readouts, we identified three FDA-approved drugs that were able to decrease CBS enzymatic activity in different cellular models. In addition, the physico–chemical properties of these molecules provide new findings on the role of copper and zinc in the regulation of CBS activity, which are crucial to develop new therapeutic avenues for the treatment of CBS-overexpressing human disorders.

## 4. Materials and Methods

### 4.1. Yeast Strains and Plasmids

*S. cerevisiae* strains used in this study are listed in Table S1 and were cultured as previously described [13]. Cultures in the exponential growth phase, obtained by diluting overnight cultures and incubating for 4–5 h to reach OD_600_~0.6–1, were used in all experiments. Yeast deletion of *MAC1* in the W303 background was performed by standard one-step gene replacement with PCR-generated cassettes [56] using primers listed in Table S2.

Subcloning of *CYS4*-FL, *CYS4*-ΔC and Ctr1Δ300 in expression vectors of the pRS42X series and full-length human CBS in the pcDNA3 vector (Invitrogen, Waltham, MA, USA) was performed as previously described [13] using primers listed in Table S2. To obtain a sufficient level of methionine auxotrophy, *CYS4* overexpression was obtained through the transfection of two different vectors of the pRS42X series, and serine was added in the medium at a final concentration of 1.5 mM.

### 4.2. Western Blot

For yeast protein extracts, 5 mL of 0.8–1.0 OD_600_ exponentially growing cells was collected, and cell pellets were resuspended into 300 μL of lysis buffer (25 mM Tris–HCl pH 6.8, 10% glycerol, 5% β-mercaptoethanol, 5% SDS, 8 M Urea, 0.02% bromophenol blue). Protein was extracted by mechanical disruption with sterile glass beads; samples were then centrifuged, and the resulting supernatants were harvested.

For HepG2 transfection, 250,000 cells were seeded per well in 6-well plates 24 h before transfection. Cells were then transfected with either the pcDNA3 vector alone (Invitrogen, Paisley, UK) or pcDNA3 vector containing human full-length CBS cDNA using JetOptimus transfection reagent (Polyplus transfection, Illkirch, France) following the manufacturer’s instructions. Transfected cells were harvested 48 h after transfection in the following buffer: 150 mM NaCl, 1% IGEPAL, 50 mM Tris–HCl pH 7.4 with protease inhibitor cocktail (Roche, Manheim, Germany). Cell lysis was then performed by 6 cycles of vigorous vortexing and freeze–thawing. Protein amount in the supernatants was evaluated by classical Bradford method.

Fifty micrograms of each sample was then loaded onto 10% NuPAGE Bis–Tris gels (precast NuPAGE, Invitrogen) and transferred onto 0.45 μm nitrocellulose membranes (GE Healthcare, Little Chalfont, UK). Membranes were blocked after 1 h at room temperature in 1X PBS containing 0.1% Igepal and 5% milk and then incubated overnight at 4 °C with the following primary antibodies: anti-CBS goat monoclonal antibody (sc46830, Santa Cruz, Dallas, TX, USA, 1:200), anti-GAPDH mouse monoclonal antibody (ab125247, Abcam, Amsterdam, The Netherland, 1:5000) or anti-α-tubulin rat monoclonal antibody (ab125247, Abcam, 1:3000). The following day, membranes were washed with fresh 1X PBS with 0.1% Igepal and incubated for 45 min with rabbit anti-goat (Southern Biotech, Birmingham, AL, USA) or goat anti-mouse secondary antibodies (Dako, Santa Clara, CA, USA) conjugated to horseradish peroxidase at a 1:3000 dilution and analysed by enhanced chemiluminescence using a Vilber–Lourmat Photodocumentation Chemistart 5000 imager (ECL, GE Healthcare, Amersham, UK).

### 4.3. Determination of Cytosolic pH

Yeast strains were transformed with a pRS416-*ADH* plasmid containing a pH-sensitive ratiometric GFP variant named pHluorin (kindly obtained from S. Léon, IJM, Paris) [57,58]. This pHluorin is only expressed in the cytosol and nucleus; it is excluded from the vacuole. A few µL of culture in the exponential growth phase was deposited on a glass slide, and yeast cells were then imaged on an epifluorescence microscope (Axio Imager M2, Carl Zeiss, Germany). Phase contrast and fluorescent images were collected with the 63× objective white brightfield channel and the green fluorescence channel (Ex/Em = 488/509 nm) with an exposure time of 500 ms. Image analysis was performed with the Zen 2.3 SP1 software (Carl-Zeiss Microcopy GmbH, Jena, Germany).

For quantitative analysis, the OD_600_ of culture in the exponential growth phase was measured. Subsequently, 10 mL of culture was centrifuged for 10 min at 3500 rpm. Cells were then resuspended in 500 μL of fresh minimal medium (MML, prepared with 6.7 g/L yeast nitrogen base without amino acids or bases). Aliquots of 100 μL of each culture were placed in triplicate in a black 96-well plate with clear bottom and read with a Varioskan LUX multimode microplate reader (Thermofisher, Marsiling Singapore) at two excitation wavelengths (410 nm and 470 nm) and a fixed emission wavelength (508 nm) for quantitative measurement, which has previously been shown to be proportional to cytosolic pH [57,59].

The resulting values were then used to calculate the I_410_/I_470_ ratio, which was expressed as a percent of the corresponding controls. Tests with drugs were performed by adding the indicated final concentrations of drugs or the corresponding volume of DMSO as a control in culture media during the growth phase for 3–4 h. OD measures were taken at the end of incubation to ensure that drugs were not toxic at tested concentrations and that pHluorin value changes could not be attributed to variations in cell amounts.

### 4.4. Cell Culture and Drug Treatment

The human liver cancer cell line HepG2 and human colon cancer cell line HCT116 were obtained from ATCC. The human dermal fibroblasts from DS subjects used here (cell line 9-FCYPR10000497) were kindly obtained from the Institut Jerôme Lejeune (Paris, France) (CODECOH declaration: DC-2019-3836). HepG2 cells and fibroblasts were cultured in DMEM glutamax high glucose medium (Invitrogen) supplemented with 10% foetal bovine serum and 100 U/mL penicillin/streptomycin (Invitrogen), whereas HCT116 cells were grown in DMEM-F12 supplemented with 5% foetal bovine serum and 100 U/mL penicillin/streptomycin (Invitrogen) in a humidified incubator at 37 °C and 5% CO_2_ atmosphere.

All the molecules used in this study were purchased from Sigma-Aldrich (St. Louis, MI, USA) except nitroxoline, which was obtained from Santa Cruz, and PBT2, which was synthesized by F. Bihel. All molecules were resuspended in DMSO. For drug treatment, 20,000 HepG2 cells, fibroblasts or 7500 HCT116 cells were plated in each well of a Greiner Bio-One black 96-well plate with transparent flat bottom in 100 µL of culture medium. The following day (HepG2 cells) or 48 h later (HCT116), cells were incubated for 24 h with selected drugs at a final concentration of 1% DMSO (*v*/*v*).

### 4.5. Mass Spectrometry Quantification of Amino Acids

A total of 250,000 cells seeded per well in 6-well plates were incubated for 24 h with the tested drugs at a final concentration of 1% DMSO (*v*/*v*). Cells were then harvested in the following buffer: 150 mM NaCl, 1% IGEPAL, 50 mM Tris–HCl pH 7.4 with protease inhibitor cocktail (Roche). Cell lysis was then performed by 10 cycles of vigorous vortexing and freeze–thawing. The protein amount of the supernatant was evaluated by the classical Bradford method.

Cystathionine, methionine, *d*_3_-cysteine, [^13^*C*, *d*_3_]-methionine, tris(2-carboxyethyl)phosphine (TCEP) and acetyl chloride were purchased from Sigma Aldrich (Saint-Quentin Fallavier, France). UPLC/MS-grade methanol, water, formic acid, hydrochloric acid solution (HCl, 1 M) and butanol were purchased from Biosolve (Valkenswaard, The Netherlands). Analyses were performed by liquid chromatography–tandem mass spectrometry (LC–MS/MS) on a Xevo^®^ Triple-Quadrupole mass spectrometer with an electrospray ionization interface equipped with an Acquity H-Class^®^ UPLC™ device (Waters Corporation, Milford, MA, USA). Individual stock solutions (10 mmol/L) of labelled and unlabelled amino acids were prepared in 0.1 M HCl. A pool of unlabelled standard solutions was prepared and serially diluted in water to obtain seven standard solutions ranging from 0.01 to 100 µmol/L. A solution of labelled internal standards (IS solution) consisting of *d*_3_-cysteine and [^13^*C*, *d*_3_]-methionine was prepared in water. The standard solutions and cell lysate samples (20 µL) were then extracted with 100 µL of methanol and 50 µL of the IS solution. The samples were mixed and centrifuged at 10,000× *g* at 10 °C for 15 min to remove the precipitated proteins. The supernatants were collected and dried under a gentle stream of nitrogen (45 °C). The derivatization step was performed by dissolving the dried extract in 100 µL of a freshly prepared butanol solution containing 5% acetyl chloride and kept at 60 °C for 30 min. The solvent was then removed under a gentle stream of nitrogen (60 °C). The dried samples were dissolved in 100 µL of water containing 0.1% formic acid and 10 mmol/L TCEP (to reduced the disulphide bond of cysteine) and injected into the LC-MS/MS system. Samples (10 µL) were injected onto an Acquity BEH-C_18_ column (1.7 µm; 2.1 × 100 mm, Waters Corporation) held at 60 °C, and compounds were separated with a linear gradient of mobile phase B (0.1% formic acid in methanol) in mobile phase A (0.1% formic acid in water) at a flow rate of 400 µL/min. Mobile phase B was kept constant at 1% for 0.5 min, linearly increased from 1% to 95% for 4.5 min, kept constant for 1 min, returned to the initial condition over 0.5 min, and kept constant for 1.5 min before the next injection. Targeted compounds were then detected by the mass spectrometer with the electrospray interface operating in positive ion mode (capillary voltage, 3 kV; desolvatation gas (N_2_) flow, 650 L/h; desolvatation gas temperature, 350 °C; source temperature, 120 °C). Multiple reaction monitoring mode was applied for MS/MS detection, as detailed in Table S3.

### 4.6. Measurement of H_2_S Production in Live Cells

Following a 24 h treatment, cells were washed once with 1X PBS and incubated for 2 h in a saline buffer (139 mM NaCl, 0.56 mM MgCl_2_, 10 mM Hepes, 2.7 mM KCl, 1 mM K_2_HPO_4_, 1.8 mM CaCl_2_ pH7.4 supplemented with 10 mM glucose) containing 100 µM of 7-azido-4-methylcoumarin (AzMC) fluorescent probe (Sigma), which selectively reacts with H_2_S to form a fluorescent compound. Fluorescent AzMC signal acquisition (λ_Ex_ = 365 nm and λ_Em_ = 450 nm) was performed on a Flexstation 3 microplate reader using SoftMax Pro 5.4.5 software (Molecular Devices, San Jose, CA, USA). Values were expressed as a percent of the corresponding controls.

### 4.7. Cell Viability Assessment

The cytotoxicity of all tested compounds was examined using a cell counting kit (WST-8/CCK8, Abcam). Briefly, following measurement of H_2_S levels, cells were washed once with PBS and incubated for 2 h in the WST-8 reagent mixed in the culture medium according to the manufacturer’s instructions. Absorbance signal acquisition (at 450 nm) was performed on a Flexstation 3 microplate reader with SoftMax Pro 5.4.5 software (Molecular Devices, San Jose, CA, USA). Values were expressed as a percent of the corresponding controls.

### 4.8. Measurement of H_2_S Production in Cell Lysates

HepG2 transfection and cell lysis were performed as described above. The equivalent of 50 µg of total proteins was then incubated with CBS substrates (10 mM cysteine and 10 mM homocysteine) and with 25 µM of the fluorescent probe AzMC diluted in 100 mM Tris–HCl pH 8 along with 50 µM of the tested compound for 2 h. AzMC fluorescent signal acquisition (λ_Ex_ = 365 nm and λ_Em_ = 450 nm) was performed on a Flexstation 3 microplate reader with SoftMax Pro 5.4.5 software (Molecular Devices, San Jose, CA, USA).

### 4.9. Measurement of HCT116 Cell Proliferation and Survival

Live-cell analysis using an Incucyte S1 was used to determine cell proliferation and survival of HCT116 cells treated with CQ, CHX, NHX or AOAA. HCT116 cells were treated with the drugs as described above, except Incucyte cytotox dye (Sartorius, Essen Bioscience, Royston, UK) was added at the same time as the drugs. Phase contrast and fluorescent images were collected with the 20× lens every 2 h for 48 h using phase contrast of the white light channel and the green fluorescence channel (300 ms exposure). Cell proliferation was assessed by determination of cell confluency analysed on the phase-contrast images captured every 2 h. Toxicity was assessed as the intensity of fluorescence of cells loaded with cytotox dye normalized to confluency at each measurement time point. Images were analysed and data were generated using Incucyte 2019B Rev2 analysis software, Sartorius, Goettingen, Germany).

### 4.10. Measurement of Reactive Oxygen Species (ROS) Level in HepG2 Cells

Cells were plated in Greiner Bio-One (Frickenhausen, Germany) black 96-well plates with transparent flat bottoms and treated as previously described for H_2_S production measurement. Reactive oxygen species (ROS) levels in HepG2 cells were monitored using the cell-permeable reagent 2′-7′-dichlorofluorescein (DCFDA, obtained from Abcam), which is oxidized by ROS to form a fluorescent compound. Briefly, following drug treatment, cells were incubated with 10 µM DCFDA for 30 min. Tert-butyl hydroperoxide (TBHP, 100 µM, Sigma-Aldrich) or H_2_O_2_ were used as positive controls. Fluorescent signal acquisition (λ_Ex_ = 485 nm and λ_Em_ = 535 nm) was performed on a Flexstation 3 microplate reader with SoftMax Pro 5.4.5 software (Molecular Devices, San Jose, CA, USA).

### 4.11. Statistical Analysis

Statistical analysis was performed using GraphPad Prism software 8.0.2 (Prism, San Diego, CA, USA). Results present data obtained in a representative experiment from at least 3 independent experiments, except for amino acid dosage results, which are presented as the mean of at least 3 independent experiments.

## Data Availability

All data and yeast strains are available on request.

## Supplementary Materials

The following supporting information [60,61,62,63,64,65] can be downloaded at: https://www.mdpi.com/article/10.3390/ijms23126769/s1.
