# Supplementary material for: Identification of 8-Hydroxyquinoline Derivatives That Decrease Cystathionine Beta Synthase (CBS) Activity
Source: Int J Mol Sci. 2022 Jun 17;23(12):6769. doi: 10.3390/ijms23126769 (PMC9223588; doi:10.3390/ijms23126769)
Supplement: Supplementary file 1 [file ijms-23-06769-s001.zip › ijms-1751228-supplementary.pdf]

International Journal of
Molecular Sciences

MDPI

Supplementary Material and Methods

Quinacrine staining

Quinacrine staining experiments were performed following the protocol from Baggett [60]. Briefly, 1 mL of 0.7 OD_{600} exponentially growing cells was gently spun down for 3 to 10 min at 300 g at room temperature, and pellets were incubated on ice for 5 min. Then, cells were resuspended in 900 µL of YPD medium with 100 µL of HEPES 1 M pH 7.6 and 10 µL of quinacrine 20 mM, and resuspended in HEPES 1 M pH 7.6 (final concentration of quinacrine: 200 µM). After 30 min incubation at 29°C with agitation of 220 rpm in darkness, cells were spun down for 3 min at 300 g at room temperature. Pellets were incubated on ice for 5 min, and resuspended in 50 µL of an ice-cold solution of 2% glucose with 100 mM HEPES. Finally, cells were spotted on microscope slides and immediately (within 10 minutes) observed under an epifluorescence microscope (Zeiss Axio Imager 2 M2) with an FITC filter.

Growth on alkaline media

Cells in exponential growth phase were diluted to an OD_{600}=0.08, and 1:5 serial dilutions were spotted on solid agar-based plates containing either unbuffered YPD, YPD pH 7.5 (buffered with 50 mM MES and 50 mM MOPS and adjusted to the indicated pH with NaOH), or YPD pH 7.5 supplemented with 60 mM $CaCl_2$. Plates were then incubated at 29°C for 3 days.

Generation of CYS4 mutant plasmids

CYS4 mutant plasmids were created by site-directed mutagenesis (QuickChange Lighting, Agilent technologies, Santa Clara, California, USA) according to the manufacturer's instructions. Primers used to generate Cys4p L503Q and G247S are listed in Table S2.

Table S1. *S. cerevisiae* yeast strains used in this study.

Strain	Genotype	Source
**S288C strains**		
BY *WT*	BY4743 *MATa/α his3Δ1/his3Δ1 leu2Δ0/leu2Δ0 met15Δ0/MET15 LYS2/lys2Δ0 ura3Δ0/ura0*	Euroscarf
cys4Δ	BY4743 except *cys4::kanMX4/cys4::kanMX4*	Euroscarf
vma1Δ	BY4742 (*MATα ura3Δ0 leu2Δ0 his3Δ1 lys2Δ0*) *vma1::kanMX4*	Euroscarf
gex1Δ/gex2Δ	BY4742 except *gex1::KanMX6 gex2::HIS3*	Dhaoui et al., 2011
**W303 strains**		
W303 *WT*	*MATa leu2-3,112 trp1-1 can1-100 ura3-1 ade2-1 his3-11,15*	Lab collection
mac1Δ	*MATa leu2-3,112 trp1-1 can1-100 ura3-1 ade2-1 his3-11,15 mac1::kanMX4*	This study

Table S2. primers used in this study.

Application	Primer Sequences
MAC1 deletion in yeast	5'-CTTGTTTTAAACTTGAAACAGTCTGGTAAGTTCTTCAAGCTCTATCAGAGCG-GATCC CCGGGTTAATTAA-3'
	5'- ATTTTCATTCCTGTTGCCTCAATGTGTTTTCTATCTGTATTTAC-GTGATTGAATTCGA GCTCGTTTAAAC-3'
CYS4-FL sub-cloning	5'-CGGGATCCCGATGACTAAATCTGAGCAGCAAG-3'
	5'-GCCTCGAGTCTTATGCTAAGTAGCTCAGTAAATCC-3'
CYS4-ΔC sub-cloning	5'-CGGGATCCCGATGACTAAATCTGAGCAGCAAG-3'
	5'-GCCTCGAGTCTTACAGCTTTGAAGAGTCAAAACG-3'
Ctr1Δ300 sub-cloning	5'- CGGGATCCCGAAATGGAAGGTATGAATATGGG-3'
	5'- GCCTCGAGTCCGCTTTCTGAATTTCTCTTTGG-3'
CYS4p L503Q mutant	5'-TCGTTACTAAGATGGATTTACAGAGCTACTTAGCATAAGACTC-3'
	5'-GAGTCTTATGCTAAGTAGCTCTGTAAATCCATCTTAGTAACGA-3'
CYS4p G247S mutant	5'- GACTACAAAGTTGAGGGTATTAGTTATGATTTTGTTCCTCAGG-3'
	5'-CCTGAGGAACAAAATCATAACTAATACCCTCAACTTTGTAGTC-3'
hCBS-FL sub-cloning	5'- CGGAATTCCGATGCCTTCTGAGACCCCCCA-3'
	5'- GCCTCGAGTCTCACTTCTGGTCCCGCTCC-3'

Table S3. Multiple reaction monitoring (MRM) transitions used for LC-MS/MS detection.

Compound	MRM Transition (*m/z*)	Cone/Collision (V)	Internal Standard (IS)
Cystathionine	335.1 → 190.0	30/15	IS1
Methionine	206.2 → 103.9	30/15	IS2
d_3-cysteine (IS1)	181.1 → 78.9	30/15	-
[^{13}C, d_3]-methionine (IS2)	210.2 → 107.9	30/15	-

Table S4. Effects of analogues of CQ and CHX on growth rescue of *CYS4*-OE cells on a methionine-free medium and on H_2S production in HepG2 cells.

Compounds	Structure	Growth Rescue 5 nmol	Growth Rescue 50 nmol	H_2S Synthesis (HepG2)
**Compound 1**<br>8-Hydroxy-5-nitro-quinoline = Nitroxoline	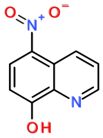	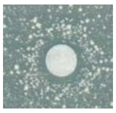<br>Positive	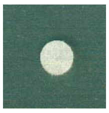<br>Toxic	≈ 30% decrease at 10 µM, ≈ 40% decrease at 20 µM
**Compound 2**<br>Chloroxine	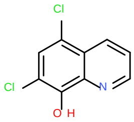	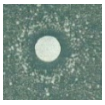<br>Positive	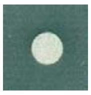<br>Toxic	≈ 15% decrease at 10 µM, ≈ 25% decrease at 20 µM
**Compound 3**<br>Clioquinol	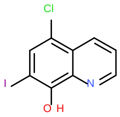	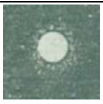<br>Positive	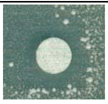<br>Toxic/positive	≈ 10% decrease at 10 µM, ≈ 20% decrease at 20 µM
**Compound 4**	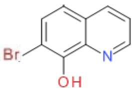	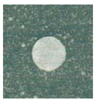<br>positive	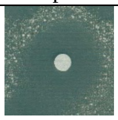<br>Toxic/positive	≈ 20% decrease at 10 µM, ≈ 30-40% decrease at 20 µM
**Compound 5**<br>5,7-Dibromoquinoline	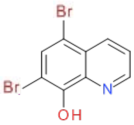	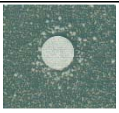<br>Positive	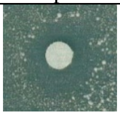<br>Toxic/slightly positive	≈ 15% decrease at 10 µM, ≈ 20% decrease at 20µM
**Compound 6**<br>5-Bromoquinolin-8-ol	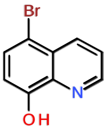	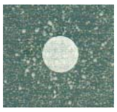<br>Positive	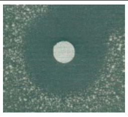<br>Toxic/positive	≈ 5% decrease at 10 µM, ≈ 15% decrease at 20 µM
**Compound 7**<br>5-Chloroquinolin-8-ol	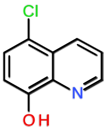	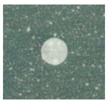<br>Slightly positive	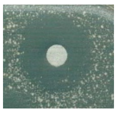<br>Toxic/slightly positive	≈ 2-3% decrease at 10 µM, ≈ 10% H_2S decrease at 20 µM
**Compound 8**<br>8-Hydroxyquinoline	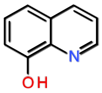	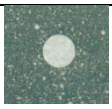<br>Slightly positive	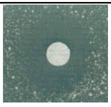<br>Toxic/slightly positive	≈ 2-3% decrease at 10 µM, ≈ 10% H_2S decrease and decreased viability at 20 µM
**Compound 9**<br>5,7-Dichloroquinoline	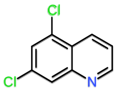	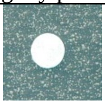<br>Negative	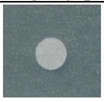<br>Negative	inactive
**Compound 10**<br>2,4-Dichloro-1-naphthol	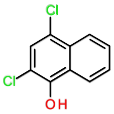	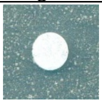<br>Negative	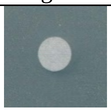<br>Negative	inactive
**Compound 11**<br>5-Amino-8-hydroxy-quinoline	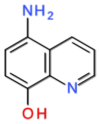	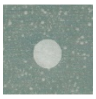<br>Negative	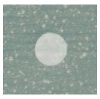<br>Negative	inactive

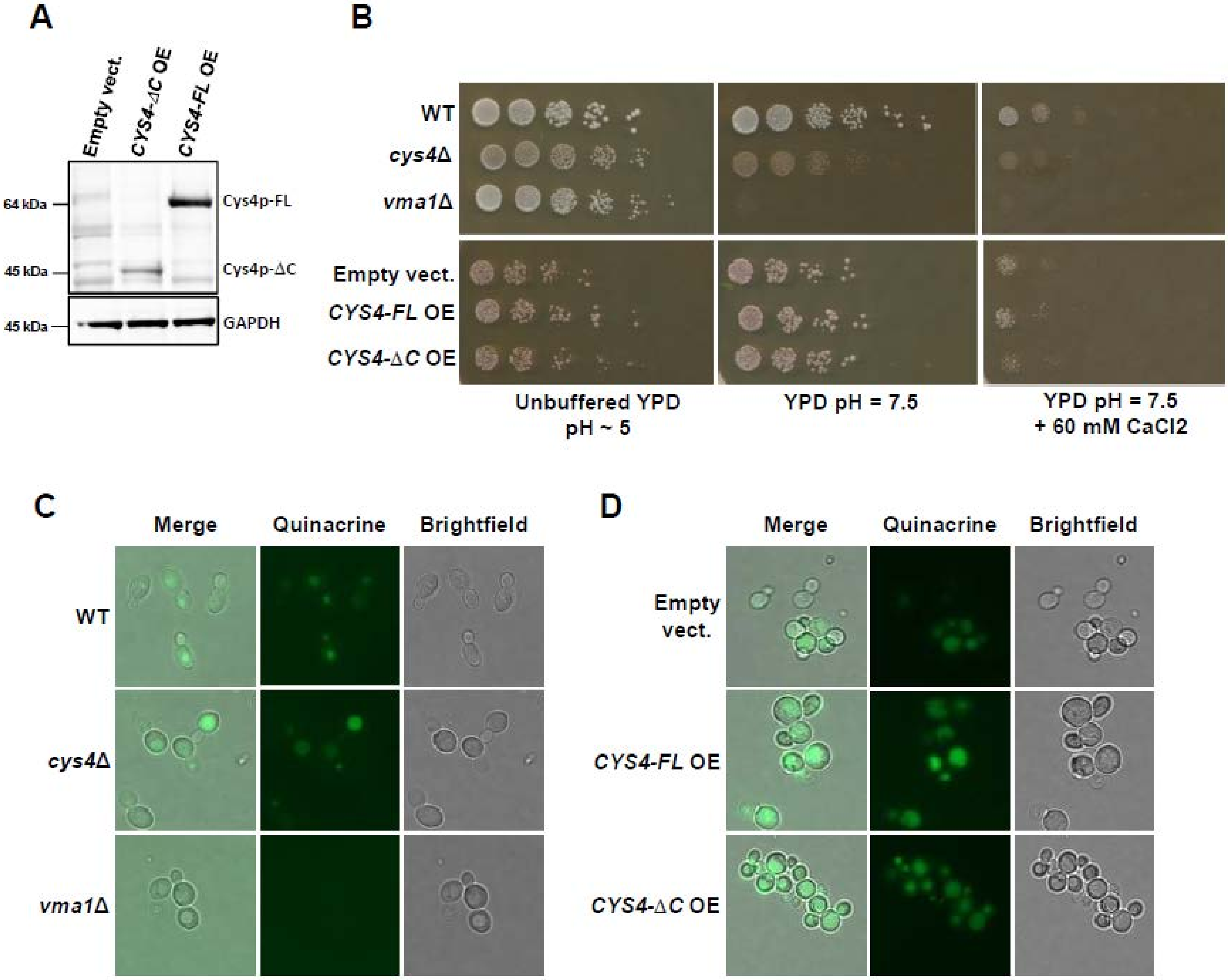

Figure S1. Impact of the modification of *CYS4* expression levels on vacuolar pH. (A) Western-blot showing the expression of either full-length (*CYS4*-FL) or the C-terminal truncated form of Cys4p (*CYS4*-ΔC) under the control of the strong GPD promoter. Two different plasmids were used to express *CYS4*-FL whereas only one plasmid was used to express *CYS4*-ΔC. Full length Cys4p was detected at approximately 64 kD whereas the C-terminal-deleted protein was detected at approximately 45 kD. GAPDH was used as a loading control. (B) Assessment of the effect of *CYS4* deletion (upper panel) or overexpression (lower panel) on yeast cell growth on alkaline medium. Cell growth was assessed on alkaline medium (pH = 7.5, medium panel) with or without high calcium concentrations (60 mM $CaCl_2$, right panel), which is commonly impaired in vacuolar acidification mutants such as *S. cerevisiae vma1*Δ that we used as a control. *VMA1* encodes the subunit A of the V1 peripheral membrane domain of the V-ATPase. Whereas control *vma1*Δ cells, which are known to entirely lack vacuolar acidification due to the complete inactivation of the V-ATPase, showed severe growth defect on alkaline medium (pH = 7.5), *cys4*Δ cells had only slight growth defect in these conditions, suggesting that vacuolar acidification may be only partially impaired in *cys4*Δ cells. Similarly, overexpression of *CYS4-FL* and *CYS4*-ΔC in wild-type cells (lower panel) did not show any different growth on alkaline medium (pH = 7.5, medium panel) and in the presence of high calcium concentrations (60 mM $CaCl_2$, right panel), compared to the control. (C-D) Vacuolar acidification is not significantly altered in *cys4*Δ and *CYS4*-OE cells as assessed by quinacrine staining. The lysosomotropic fluorescent dye quinacrine, which normally accumulates in acidic vacuoles, was used to assess the effect of *CYS4* deletion or overexpression on vacuolar acidification. (C) *cys4*Δ cells revealed an intermediate phenotype with less quinacrine staining (indicative of slightly less acidified vacuoles) than a wild-type (WT) strain, but more than the *vma1*Δ strain. This suggests that vacuolar acidification is only slightly impaired in *cys4*Δ cells. This is in agreement with what has been described by Sambade [61]who found a less severe phenotype of *cys4*Δ than the one originally described by

Oluwatosin and Kane [21]. (D) Conversely, overexpression of both *CYS4-FL* and *CYS4-ΔC* in a wild-type strain induced slightly increased quinacrine staining, suggestive of a slight increase in vacuolar acidification. Two different plasmids were used to express *CYS4*-FL whereas a single plasmid was used to overexpress *CYS4*-ΔC. To conclude, modifications in the level of expression of *CYS4* only have a slight effect on vacuolar acidification making the vacuolar pH modifications resulting from *CYS4*-OE a too subtle phenotype to be used as a robust readout for drug or genetic screening.

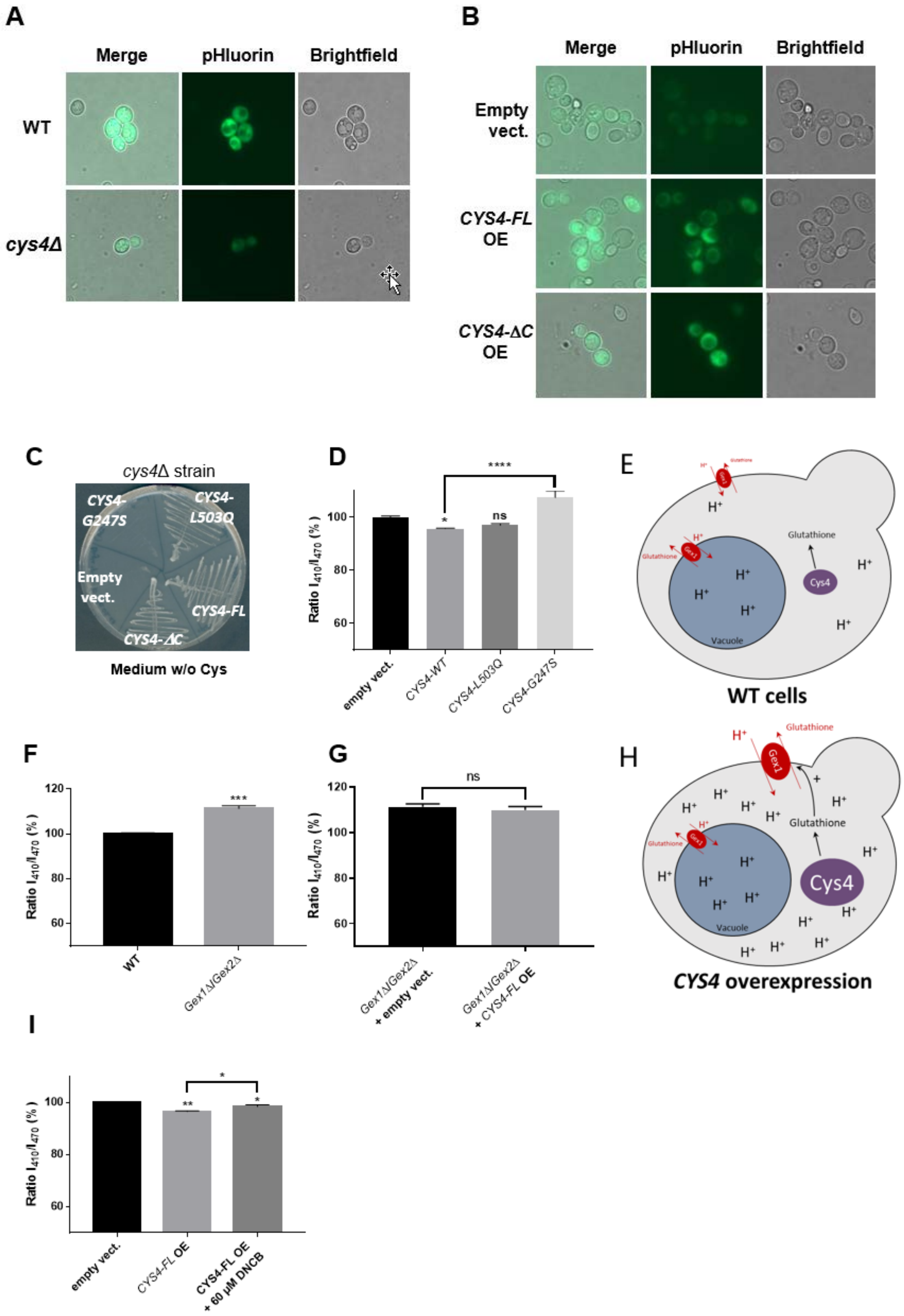

Figure S2. *CYS4* expression levels have an impact on cytosolic pH homeostasis. (A) Effect of *CYS4* deletion on cytosolic alkalinization assessed by pHluorin-dependent fluorescence intensity. *cys4*Δ cells displayed a strong decrease in cytosolic pHluorin signal compared to a wild-type (WT) strain, indicating that *cys4*Δ cells have a more alkaline cytosolic environment than wild-type cells. (B) Effect of *CYS4*-OE on cytosolic acidification assessed by pHluorin-dependent fluorescence intensity. Wild-

type cells overexpressing either forms of Cys4p (full-length or deleted of its C-terminal domain) led to a significant increase in fluorescence intensity, suggestive of a more acidic cytosolic pH. Note that the exposure time settings of the fluorescent microscope were different between (A) and (B) to make differences with *Cys4*Δ or *CYS4*-OE more visible on the pictures. (C-D) Cytoplasmic acidification is directly linked to Cys4p enzymatic activity. Two *CYS4* mutants corresponding to two different *CBS* mutations found in patients with homocystinuria were used to investigate the link between Cys4p enzymatic activity and cytoplasmic acidification. The mutation p.L503Q in Cys4p, corresponding to p.L540Q in CBS, has been reported to have enzymatic activity in the range of the WT protein but is insensitive to the regulation by S-adenosylmethionine (SAM) [62], a metabolite that activates CBS to adjust its activity to methionine levels. The second mutation, p.G247S in Cys4p corresponding to p.G307S in CBS, is one of the most frequent mutation found in homocystinuria, and has no detectable residual enzymatic activity [63]. In agreement with the finding that Cys4p activity is not regulated by SAM in yeast [64], Cys4p mutant p.L503Q was found to be able to rescue the growth of a *cys4*Δ strain on medium without cysteine whereas the mutant p.G247S was not (C). Similarly, p.L503Q mutant was able to decrease the cytosolic pH of a *cys4*Δ strain although less efficiently than *CYS4*-WT. Conversely, p.G247S mutant did not decrease cytosolic pH, it even increased it (D). (E-I) Gex1p and Gex2p glutathione:proton exchangers are involved in the cytosolic acidification phenotype of *CYS4*-OE cells. As defects in cytosolic pH homeostasis have never been reported for *cys4*Δ or *CYS4*-OE cells, we investigated the relationship between Cys4p and two proteins known to be important for the regulation of intracellular pH in yeast, Gex1p and Gex2p [65]. These two glutathione:proton exchangers, located both at the vacuolar and plasma membrane, extrude glutathione out of the cytosol in exchange for protons entry, thus affecting cytosolic pH homeostasis (E). As previously described [65], a yeast strain deleted for *GEX1* and *GEX2* genes has higher cytosolic pH as shown by the measurement of the I_{410}/I_{470} ratio (F). In this *gex1*Δ, *gex2*Δ double mutant strain, *CYS4*-OE was unable to induce cytosolic acidification (G), suggesting that cytosolic acidification of *CYS4*-OE cells may be partly related to increased intracellular glutathione production, which in turn activates Gex1p/Gex2p exchangers that allow proton entry into the cell (H). To further validate this hypothesis, we used 1-Chloro-2,4-dinitrobenzene (DNCB), an oxidant molecule that can decrease intracellular levels of glutathione. *CYS4*-OE cells treated with 60 μM of DNCB showed a slight but significant increased cytosolic pH compared to *CYS4*-OE (I), confirming the direct relationship between intracellular glutathione levels and cytosolic pH variations induced by the modification of *CYS4* expression levels. Altogether, these data show that *CYS4* deletion leads to increased cytosolic pH whereas *CYS4* overexpression leads to more acidic cytosolic pH. (D and I) One-way ANOVA with Tukey's post-hoc test. (F-G) Student's t-test. *ns: not statistically significant; *, p<0.05; **, p<0.01; ***, p<0.001; ****, p<0.0001.*

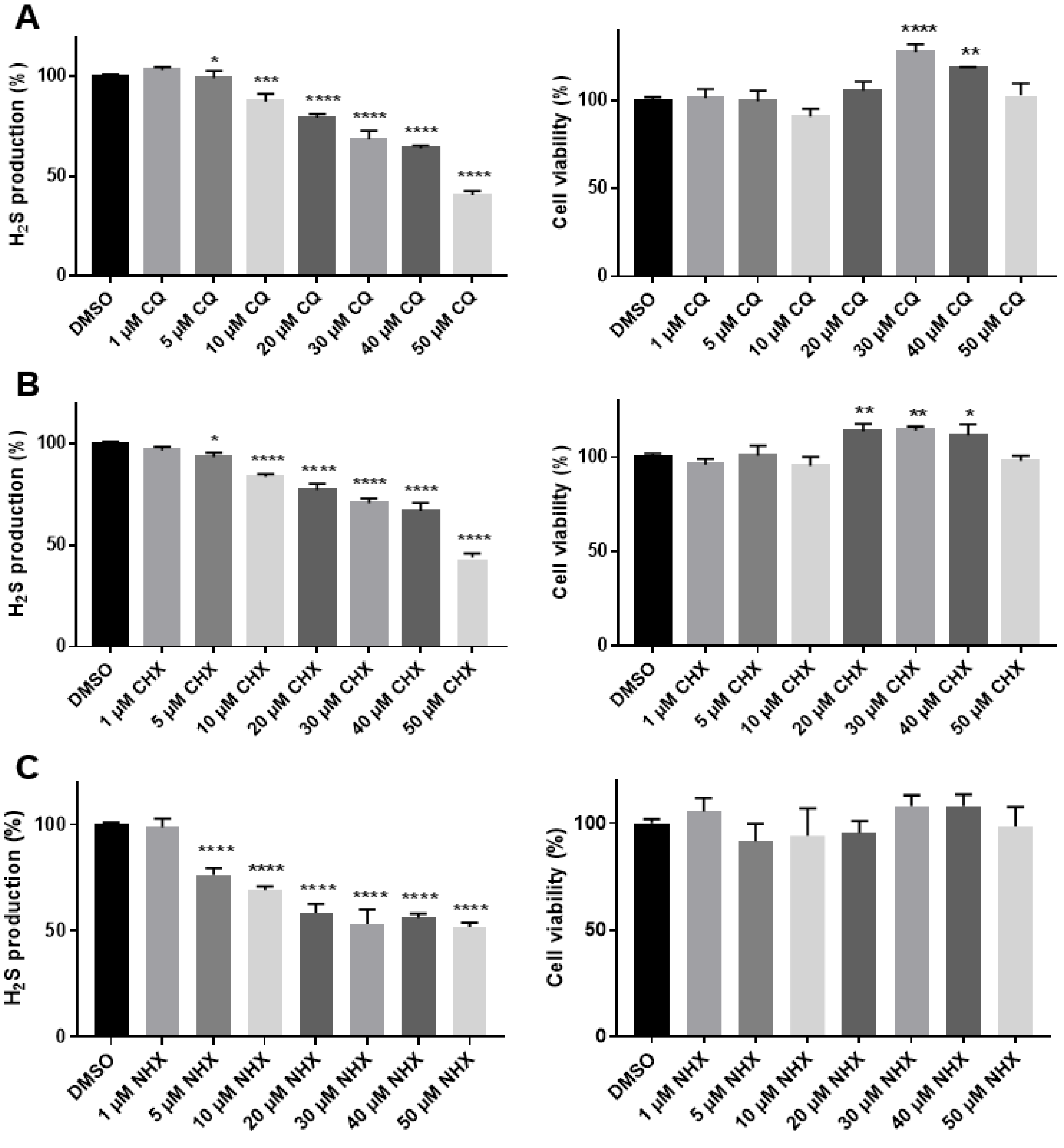

Figure S3. Effect of CQ, CHX and NHX on H_2S production in HepG2 cells. (A-C) Dose-dependent decrease of H_2S production following a 24-hour incubation of HepG2 cells with indicated amounts of CQ (A), CHX (B) and NHX (C). Cell viability was assessed by the WST-8 assay. Note that CQ and CHX have a tendency to increase cell proliferation at 30-40 µM. Comparison of each condition with DMSO, one-way ANOVA with Dunnett's post-hoc test: *, *$p<0.05$*; **, *$p<0.01$*; ***, *$p<0.001$*, ****, *$p<0.0001$*.

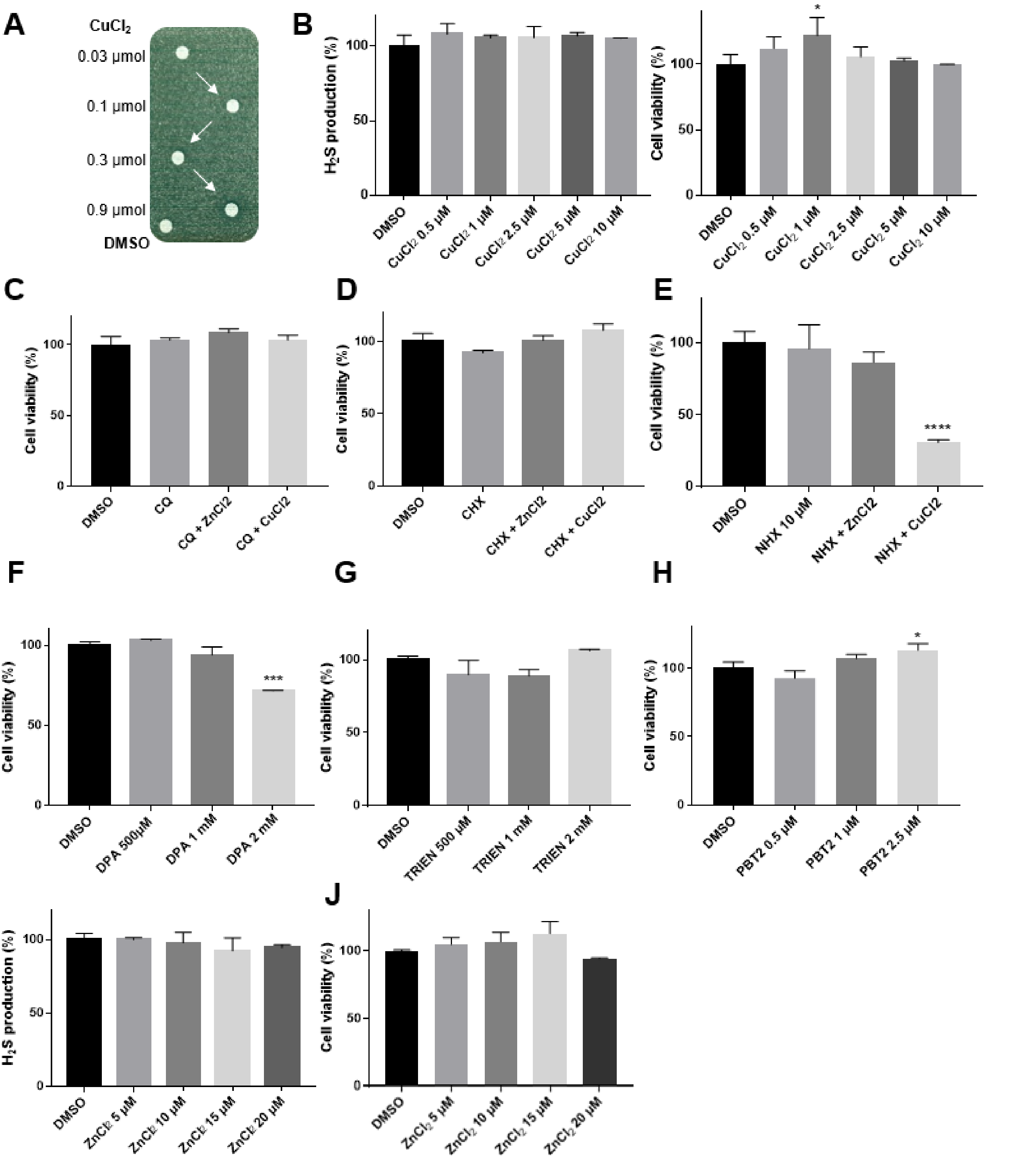

Figure S4. Effect of copper and zinc on cystathionine beta synthase activity in yeast and HepG2 cells. (A) Effect of increasing amounts of $CuSO_4$ on *CYS4*-OE cell growth. Addition of $CuSO_4$ on filters did not have any effect on *CYS4*-OE cell growth on methionine-free medium. Amounts of copper higher than 1 µmol were toxic. (B) Effect of a 24-hour treatment of HepG2 cells with increasing concentrations of $CuCl_2$. Present in the bovine serum used in cell culture, copper was estimated to be present at approximately 0.2 µM in the cell medium we used to cultivate HepG2 cells. H_2S production and cell viability were assessed as previously described using AzMC probe and WST-8 assay, respectively. Note that low amounts of copper showed a slight positive effect on cell growth. (C-E) Cell viability assessed by WST-8 assay following a 24-hour treatment of 10 µM of CQ (C), CHX

(D) or NHX (E) in combination with 10 μM of $ZnCl_2$ or 2.5 μM of $CuCl_2$. (F-H) Cell viability assessed by WST-8 assay following a 24-hour treatment of HepG2 cells with the indicated amounts of D-penicillamine (F), trientine (G) and PBT2 (H). Note that at 2 mM of DPA, decreased cell viability is observed. (I) Effect of a 24-hour treatment of HepG2 cells with increasing concentrations of $ZnCl_2$. Present in the bovine serum used in cell culture, zinc was estimated to be present at approximately 4 μM in the cell medium we used to cultivate HepG2 cells. (J) WST-8 assay following a 24-hour treatment of HepG2 cells with increasing concentrations of $ZnCl_2$.. Comparison of each condition with DMSO; one-way ANOVA with Dunnett's post-hoc test: *, *$p<0.05$*; ***, *$p<0.001$*, ****, *$p<0.0001$*.

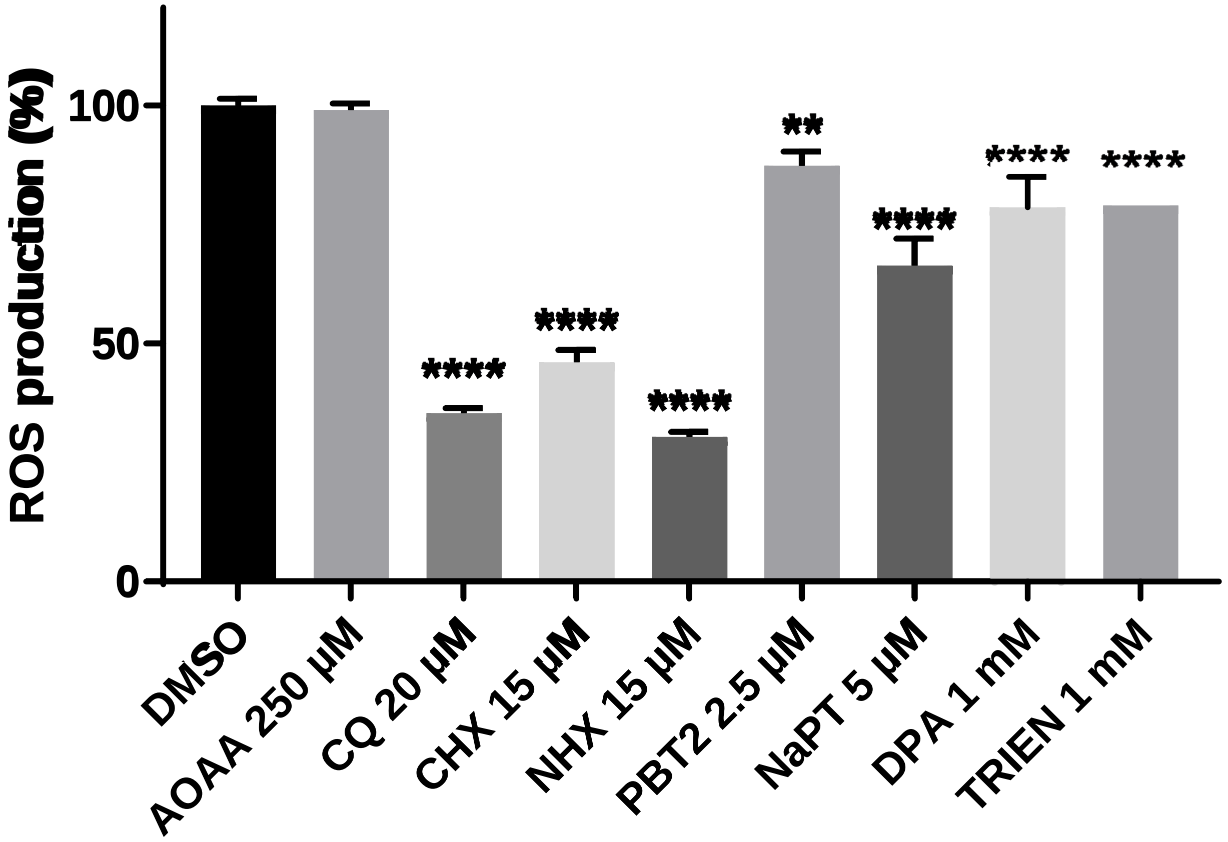

Figure S5. Effect of CQ, CHX and NHX on HCT116 cell proliferation and survival and on ROS production in HepG2 cells. (A-D) Effect of a 48-hour treatment with CQ, CHX, NHX and AOAA on HCT116 cell proliferation and survival. A 48-hour treatment of HCT116 cells with 10-50 μM of CQ (A), CHX (B) or NHX (C) decreased both cell proliferation and survival. AOAA, used as a control, also decreased cell proliferation but with much lower efficiency (D) and did not have any effect on cell survival. (E) Measure of ROS production in HepG2 cells following a 24-hour treatment with drugs at indicated concentration. Note that all drugs used at concentrations that lower H_2S production show antioxidant properties except AOAA. Comparison of each condition with DMSO; one-way ANOVA with Dunnett's post-hoc test: ** *$p<0.01$*; **** *$p<0.005$*.
